# *Opuntia ficus-indica* Flour Modulates Fecal Microbiota, Reduces Cerebral Oxidative Stress and Improves Cognitive Function in Elderly Rats

**DOI:** 10.1007/s11130-026-01510-3

**Published:** 2026-05-07

**Authors:** Renally de Lima Moura, Diego Elias Pereira, Maria da Vitória Santos do Nascimento, Larissa Maria Gomes Dutra, Roberto Germano Costa, Marcelo Sobral da Silva, Josean Fechine Tavares, Yuri Mangueira do Nascimento, Vanessa Bordin Viera, Juliano Carlo Rufino Freitas, Wydemberg José de Araújo, Fábio Anderson Pereira da Silva, Valquiría Cardoso da Silva Ferreira, Ariosvaldo Nunes de Medeiros, Juliana Kessia Barbosa Soares

**Affiliations:** 1 Food Science and Technology Program, Federal University of Paraíba, João Pessoa, PB Brazil; 2 Laboratory of Experimental Nutrition, Department of Nutrition, Federal University of Campina Grande, Cuité, Brazil; 3 Center for Education and Health, Federal University of Campina Grande, Cuité, Brazil; 4 Post-Graduate Program in Natural Sciences and Biotechnology, Center for Education and Health, Federal University of Campina Grande, Cuité, Brazil; 5 Center for Medical Sciences, Graduate Program in Translational Health, Federal University of Pernambuco, Recife, PE Brazil; 6 Department of Agriculture, Technologists Training Center - Campus IV, Federal University of Paraíba, Rio Tinto, Brazil; 7 Post-Graduate Program in Bioactive Natural and Synthetic Products, Health Sciences Center, Federal University of Paraíba, João Pessoa, Brazil; 8 Laboratory for Synthesis and Analysis of Natural Antioxidants, Department of Nutrition, Federal University of Campina Grande, Cuité, CG Brazil; 9 Education and Health Center, Academic Unit of Biology and Chemistry, Federal University of Campina Grande, Cuité, CG Brazil; 10 Federal Institute of Education, Science and Technology of Paraíba, Princesa Izabel, Brazil; 11 Chromatography and Spectrometry Laboratory, Department of Agroindustrial Management and Technology, Federal University of Paraíba, Bananeiras, Brazil; 12 Program in Agrifood Technology, Federal University of Paraíba, Bananeiras, Brazil; 13 Center for Agricultural Sciences - Campus III, Department of Animal Science, Federal University of Paraíba, Bananeiras, Brazil

**Keywords:** Gut–brain axis, Bioactive compounds, Neuroprotection, Memory and nutrition and aging

## Abstract

This study aimed to investigate the effects of *Opuntia ficus-indica* flour (OFIF) consumption on fecal microbiota composition, cognitive function, and brain oxidative stress in RI elderly male rats. Animals were randomized into five groups: Adult Control (ACG) and Elderly Control (ECG), fed the AIN-93 M diet without OFIF; and three treated groups (OF5, OF10, OF15) receiving diets supplemented with 5%, 10%, or 15% OFIF, respectively. Memory performance was assessed using non-associative learning and novel object recognition tests, with recognition (DI) and discrimination (RI) indices. Brain fatty acid profile, protein carbonyls, and glutathione levels were measured, and fecal microbiota was analyzed by 16 S rRNA amplicon sequencing. The experimental groups showed reduced locomotion and number of crossings during the second exposure to the open field test. OF15 rats spent more time in the inner zone and achieved higher DI and RI scores in both short- and long-term evaluations. Regarding oxidative stress, OF15 exhibited elevated glutathione levels, while OF5 showed reduced protein carbonyls. Treated groups displayed increased brain concentrations of oleic, eicosenoic, arachidonic, and docosahexaenoic acids. Moreover, OF15 restored intestinal microbial diversity, evidenced by higher richness indices (Chao1 and Fisher) and enrichment of short-chain fatty acid–producing genera, including *Faecalibaculum*, *Dorea*, *Prevotella*, and *Lachnospiraceae* NK4A136. In conclusion, OFIF supplementation, particularly at 15%, modulated gut microbiota, improved memory performance, and enhanced antioxidant defenses in aged rats. These results highlight OFIF as a promising dietary strategy to modulate the gut–brain axis, supporting memory preservation, oxidative balance, and intestinal health during aging.

**Supplementary Information:**

The online version contains supplementary material available at 10.1007/s11130-026-01510-3.

## Introduction

Aging is a complex and gradual process characterized by structural, physical, and chemical changes in the body and the brain. In addition to biological modifications, environmental and sociocultural factors, such as quality of life, diet, physical activity and emotional well-being, play crucial roles in the development of healthy aging [1, 2]. In recent years, a significant increase in the aging population has been observed, accompanied by a rising prevalence of chronic diseases, neurodegenerative conditions, and mental disorders [3].

Aging is associated with an increased production of reactive oxygen species (ROS) and a decline in the efficiency of endogenous antioxidant systems. This redox imbalance leads to the accumulation of free radicals, which induce oxidative damage to lipids, proteins, and deoxyribonucleic acid (DNA), particularly compromising neural function [4]. The oxidation of lipids and proteins essential for neuronal activity results in the formation of highly reactive by-products, such as carbonyl residues and malondialdehyde [5]. Protein carbonylation alters protein structure and function, thereby impairing cellular homeostasis. Malondialdehyde, in turn, disrupts cellular membranes and interferes with vital processes such as cell signaling and the integrity of the extracellular matrix. The accumulation of these by-products is strongly associated with neurological disorders, including cognitive deficits, anxiety and the progression of neurodegenerative diseases [6, 7].

Dietary antioxidants, found in significant quantities in plant-based matrices, play a crucial role in mitigating oxidative stress, thereby contributing to cellular protection and healthy aging [8]. Within the gut, these antioxidants aid in modulating the microbiota by increasing the relative abundance of health-promoting microorganisms [9]. These microorganisms, in turn, possess the ability to biotransform antioxidants into more bioavailable metabolites, which can cross the intestinal barrier, enter systemic circulation, and traverse the blood–brain barrier [10]. Once in the central nervous system, these compounds are capable of reducing oxidative stress, modulating inflammatory pathways, and enhancing neuronal signaling, thereby supporting cognitive function and exerting neuroprotective effects [11].

*Opuntia ficus-indica*, a member of the Cactaceae family, stands out as a plant-based food matrix rich in antioxidant compounds and is recognized for its notable nutraceutical potential. Adapted to extreme conditions of water stress and high temperatures, this species has garnered increasing scientific interest due to its diverse bioactive composition, including flavonoids, betalains, dietary fibers and essential fatty acids [12]. Preclinical studies in rodent models have demonstrated that these compounds exert antioxidant, anti-inflammatory, and neuroprotective effects, modulating physiological and pathological processes related to oxidative stress and inflammation [6, 13].

Despite these promising findings, there remains a gap in the literature regarding the effects of cactus consumption in aged rodents, particularly concerning the efficacy and safety of different dosages during this stage of life. Given that aging can alter the bioavailability of and physiological responses to various nutrients, we hypothesized that the bioactive compounds present in *Opuntia ficus-indica* may exert antioxidant, anti-inflammatory, and neuroprotective effects in aged organisms.

The consumption of *Opuntia ficus-indica* flour may represent a promising nutritional strategy for modulating the gut–brain axis, with potential benefits for memory improvement, intestinal microbiota balance, and reduction of cerebral oxidative stress. In light of this perspective, the present study aimed to investigate the effects of *Opuntia ficus-indica* flour consumption on fecal microbiota composition, cognitive function, and oxidative stress in the brains of aged rats.

## Materials and Methods

Supplementary material incorporated the section of Materials and methods.

## Results and Discussion

### Assessment of Non-Associative Learning

**Fig. 1:**
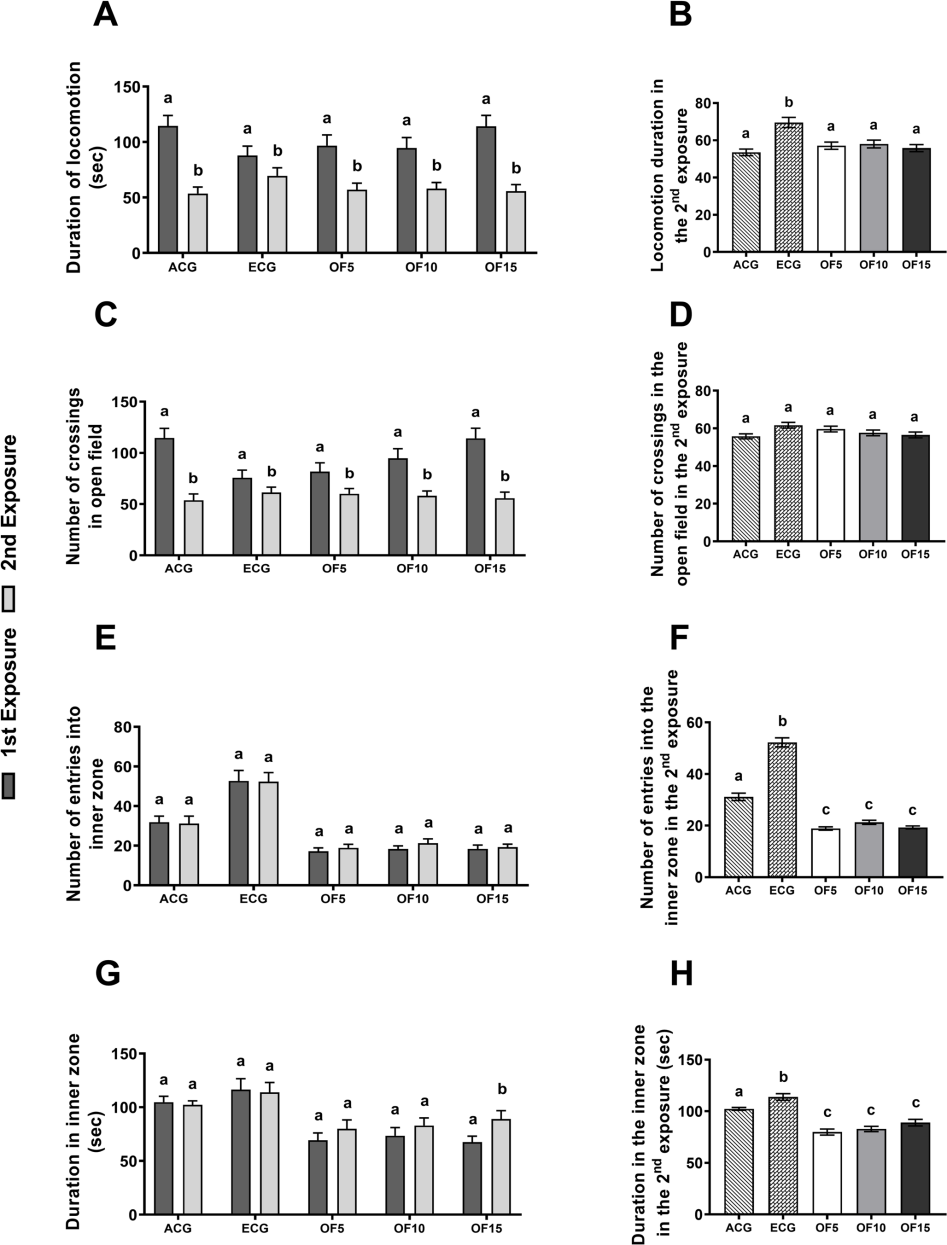
Habituation test in elderly rats treated with 5%, 10, and 15% OFIF. Data are presented as mean ± standard deviation (One-way ANOVA, Holm-Sidak). First exposure occurred at 90 days of age (adult control- ACG) and 547 days of age (elderly control and experimental groups - ECG); second exposure at 97 days (adult control - ACG) and 554 days (elderly control and experimental groups - ECG). Group sizes: ACG (Adult Control: animals fed a standard AIN-93 M diet *n* = 10), ECG (Elderly Control: animals fed a standard AIN-93 M diet *n* = 10), OF5 (animals fed a diet containing 5% cactus flour *n* = 10), OF10 (animals fed a diet containing 10% cactus flour *n* = 10), OF15 (animals fed a diet containing 15% cactus flour *n* = 10). Different letters between bars signify differences between groups (*p* < 0.05). (**A**) Locomotion duration: total time spent moving in the open field. (**B**) Locomotion duration in the second exposure: total time spent moving during the second exposure. (**C**) Number of crossings: counted when all four paws entered a segment. (**D**) Number of crossings in the second exposure: counted when all four paws entered a segment. (**E**) Number of entries into the inner zone: counted when all four paws entered the inner zone. (**F**) Number of entries into the inner zone in the second exposure: counted when all four paws entered the inner zone. (**G**) Time spent in the inner zone. (**H**) Time spent in the inner zone in the second exposure

This study evaluated the effects of *Opuntia ficus-indica* flour (OFIF) supplementation on non-associative learning in aged rats. OFIF improved behavioral performance, particularly in OF15. In the open field test, all groups showed reduced locomotion and crossings during the second exposure [F(9,78) = 85.98, *p* < 0.0001; F(9,85) = 94.18, *p* < 0.0001], indicating habituation and environmental recognition. Experimental groups exhibited lower locomotor activity than ECG, approaching ACG values [F(4,39) = 8.263, *p* < 0.0001] (Figs. 1A–C). Only OF15 showed increased time in the inner zone during the second exposure [F(4,63) = 6.341, *p* < 0.0002], suggesting that reduced locomotion reflects adaptive behavior rather than anxiety (Fig. 1H). Consistently, experimental groups showed reduced entries and time in the inner zone compared to controls (ECG and ACG) [F(4,31) = 160.3, *p* < 0.0001; F(4,32) = 27.94, *p* < 0.0001] (Figs. 1E e 1G).

These behavioral adaptations may reflect a neuroprotective response associated with bioactive compounds in OFIF, including polyphenols and unsaturated fatty acids [10]. Mechanistically, this may involve activation of the Nrf2-ARE pathway and inhibition of NF-κB, contributing to reduced oxidative stress and inflammation, supporting cognitive function during aging [14].

Locomotion duration was positively correlated with *Prevotella* (*r* = 0.82; *p* < 0.0001), while time in the inner zone (*r* = 0.91; *p* < 0.0001) and crossings (*r* = 0.62; *p* < 0.0001) were positively correlated with total brain PUFA concentration. Additionally, the RI index correlated with cerebral DHA levels (*r* = 0.82; *p* < 0.0001) Fig. 1 (available in the supplementary material). Together, these findings suggest that behavioral adaptations are associated with microbiota modulation and lipid remodeling, supporting a functional link between gut-derived signals and brain physiology.

## Object Recognition

**Fig. 2:**
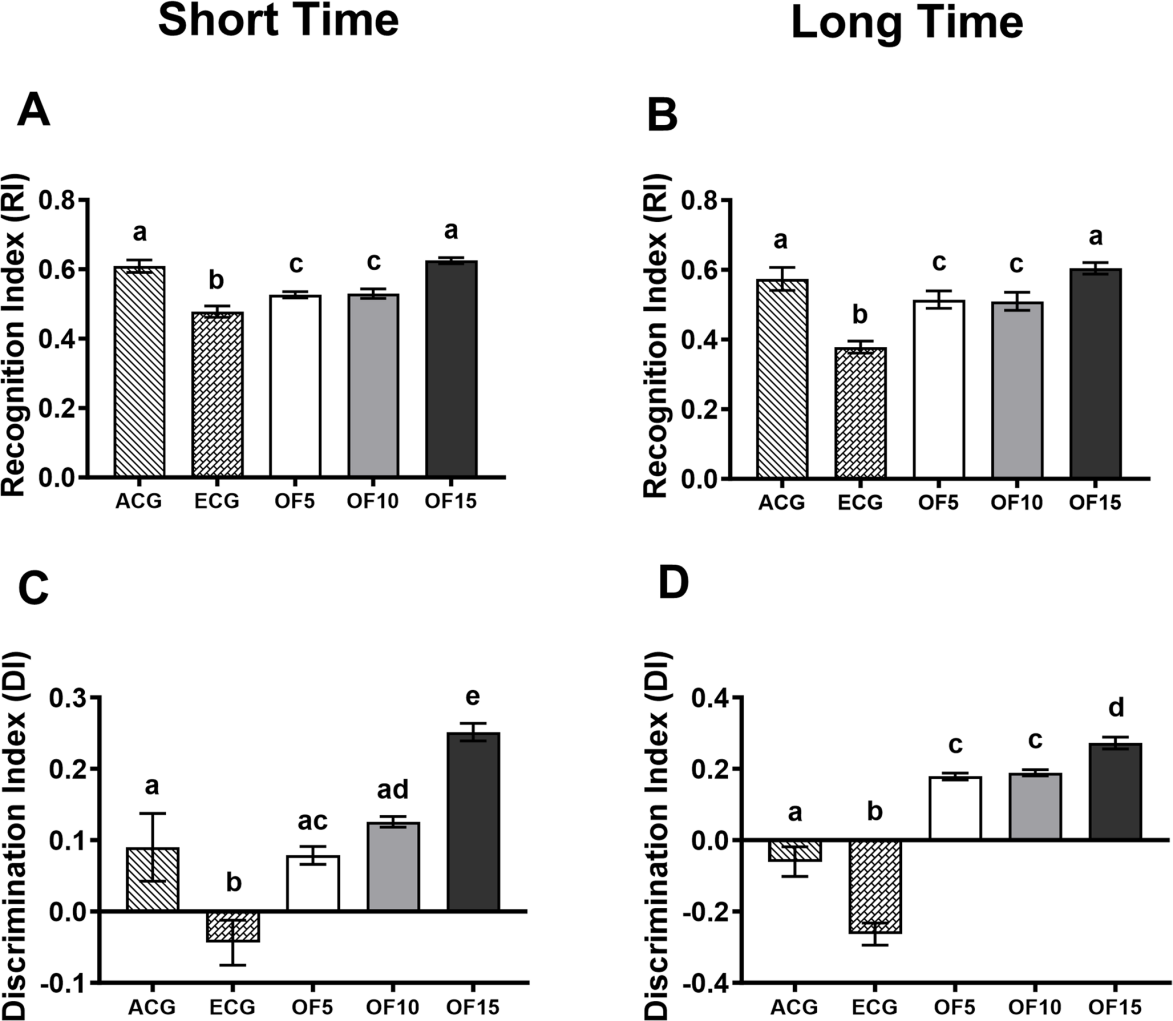
Short- and long-term object recognition test in elderly rats treated with 5, 10 and 15% OFIF. Data are presented as mean ± standard deviation (One-way ANOVA, Holm-Sidak). Group sizes: ACG (Adult Control: animals fed a standard AIN-93 M diet *n* = 10), ECG (Elderly Control: animals fed a standard AIN-93 M diet *n* = 10), OF5 (animals fed a diet containing 5% cactus flour *n* = 10), OF10 (animals fed a diet containing 10% cactus flour *n* = 10), OF15 (animals fed a diet containing 15% cactus flour *n* = 10). Different letters between bars signify differences between groups (*p* < 0.05). (**A**) Recognition Index (Short Time). (**B**) Recognition Index (Long Time). (**C**) Discrimination Index (Short Time) (**D**) Discrimination Index (Long Time)

Data from the NOR test showed improved cognitive performance in all OFIF-treated groups compared to ECG. The OF15 group exhibited the highest exploration of the novel object in both short-term (0.63 ± 0.02) and long-term (0.60 ± 0.05) tests, with values comparable to ACG (short-term: 0.61 ± 0.06; long-term: 0.57 ± 0.09) (Fig. 2A and B). No differences were observed between OF5 and OF10. In contrast, ECG showed lower recognition (RI) and discrimination (DI) indices, indicating impaired recognition memory, consistent with age-related hippocampal dysfunction and reduced neuroplasticity [15].

In the short-term test, DI was higher in OF15 (ACG: 0.09 ± 0.14; ECG: − 0.04 ± 0.09; OF5: 0.08 ± 0.04; OF10: 0.14 ± 0.03; OF15: 0.24 ± 0.04; *p* < 0.0001) (Fig. 2C). Similarly, in the long-term test, OF15 showed the highest positive DI (ACG: − 0.06 ± 0.12; ECG: − 0.26 ± 0.10; OF5: 0.18 ± 0.04; OF10: 0.19 ± 0.04; OF15: 0.27 ± 0.05; *p* < 0.0001) (Fig. 2D), indicating preserved recognition memory. RI and DI values in OF15 were comparable to ACG, supporting improved memory performance. 

These cognitive effects further support a multifactorial neuroprotective action involving antioxidant and anti-inflammatory properties of bioactive compounds, such as polyphenols and unsaturated fatty acids [16, 17]. Mechanistically, this may involve activation of the Nrf2-ARE pathway and inhibition of NF-κB, reinforcing the role of redox balance in cognitive preservation during aging [14].

RI was positively correlated with *Faecalibaculum* (*r* = 0.72), *Prevotella* (*r* = 0.97), and *Lachnospiraceae* (*r* = 0.92) (*p* < 0.0001), as well as with total brain fatty acid concentration (*r* = 0.89; *p* < 0.0001). DI also correlated positively with *Lachnospiraceae* (*r* = 0.94; *p* < 0.0001) Fig. 1 (available in the supplementary material), indicating that cognitive improvements were associated with microbiota modulation and changes in brain lipid composition.

## Fatty Acid Profile in Brain Tissue

Table 1, available in the supplementary material, shows the concentrations of fatty acids present in the brains of the animals. Among saturated fatty acids (SFA), palmitic acid levels were reduced in treated groups compared to ACG (*p* < 0.05), with the lowest levels in OF5 (*p* < 0.05). OF10 and OF15 did not differ from ECG (*p* > 0.05), although both remained lower than ACG (*p* < 0.05). Stearic acid was also reduced in OF5 compared to all groups (*p* < 0.05), while OF10 and OF15 did not differ. Total SFA was significantly lower in OF5 (*p* < 0.05).

Among MUFA, oleic acid increased in treated groups (*p* < 0.05), with no differences among OF5, OF10, and OF15 (*p* > 0.05). Eicosenoic acid was detected in all groups (ACG: 1.45 ± 0.01), peaking in OF5 (4.52 ± 0.01), while OF10 (1.18 ± 0.02) was similar to ACG, and OF15 (3.12 ± 0.03) did not differ from ECG (2.48 ± 0.02). Total MUFA was higher in supplemented groups, particularly OF5 and OF15 (*p* < 0.05).

Regarding PUFA, linoleic acid was reduced in OF10 (0.56 ± 0.01) and OF15 (0.54 ± 0.01) compared to ACG (*p* < 0.05), while OF5 showed higher levels (0.72 ± 0.02; *p* < 0.05). In contrast, arachidonic acid and DHA increased with supplementation, with highest levels in OF15 (*p* < 0.05). Total PUFA was also higher in OF15 (*p* < 0.05). ω3 increased progressively, whereas ω6 decreased compared to ACG but remained higher than ECG (*p* < 0.05), resulting in a reduced ω6/ω3 ratio, lowest in OF15 (*p* < 0.05).

DHA levels were positively correlated with *Faecalibaculum* (*p* < 0.0001; *r* = 0.84), while total PUFA correlated with *Prevotella* (*p* < 0.0001; *r* = 0.97) and *Lachnospiraceae* (*p* < 0.0001; *r* = 0.84), as well as with PWY-5484 (*p* < 0.0001; *r* = 0.63) Fig. 1 (available in the supplementary material). Notably, OF15 showed increased oleic, eicosenoic, arachidonic (ARA), and docosahexaenoic acids (DHA), which support membrane integrity, synaptic function, and neurogenesis [18–20], and may underlie improved cognition, while the reduced ω6/ω3 ratio suggests a less pro-inflammatory lipid profile [21].

Although brain lipid composition is relatively stable during aging, *Opuntia ficus-indica* flour promoted lipid remodeling. Despite its low lipid content, it provides PUFA precursors, particularly linoleic (C18:2 n-6) and α-linolenic acids (C18:3 n-3), which may support endogenous synthesis of ARA and DHA [22]. This process likely involves phospholipid turnover and enzymatic remodeling (e.g., phospholipases and acyltransferases), influenced by precursor availability and transport across the blood–brain barrier [23, 24].

The absence of significant differences in intermediate groups (*e.g*., OF10 vs. ECG or ACG) suggests a threshold effect, where moderate supplementation is insufficient to induce substantial changes. While SFA showed partial modulation, DHA exhibited a clear dose-dependent increase, reflecting its selective incorporation into neuronal membranes. These findings indicate dose-dependent and non-linear effects of *O. ficus-indica* on brain lipid metabolism.

*Opuntia ficus-indica* presents a complex nutritional and phytochemical profile that may explain the biological effects observed. Its cladodes are rich in carbohydrates, particularly soluble and insoluble dietary fibers, which modulate gut microbiota composition and metabolic activity, contributing to gut–brain axis regulation [13]. Additionally, microbiota modulation may act as a complementary mechanism, as fermentable components of *O. ficus-indica* enhance the production of short-chain fatty acids (SCFAs), such as acetate, propionate, and butyrate [25]. These metabolites regulate systemic lipid metabolism via AMPK and PPAR pathways, influencing the availability of circulating lipid precursors. Furthermore, SCFAs exert anti-inflammatory effects, reduce neuroinflammation, and may modulate gene expression through epigenetic mechanisms, supporting the preservation of polyunsaturated fatty acids and lipid remodeling in the brain [26].

Polyunsaturated fatty acids are highly susceptible to oxidation, particularly in the brain due to its high metabolic activity and continuous ROS production [27]. Thus, maintaining a balance between lipid enrichment and antioxidant protection is essential. The observed effects likely reflect not only changes in lipid composition but also a more favorable redox environment, supported by bioactive compounds from *Opuntia ficus-indica*, reinforcing its neuroprotective potential.

Aging is associated with increased ROS and reduced antioxidant defenses, promoting lipid peroxidation and neuronal damage [6]. In this context, dietary antioxidants have been linked to cognitive preservation, particularly in aged models [13, 28]. *O. ficus-indica* cladodes are rich in phenolics and flavonoids, including p-coumaric and chlorogenic acids, rutin, and isorhamnetin derivatives, which exhibit antioxidant and anti-inflammatory properties. Their high phenolic content and antioxidant activity (FRAP and ABTS) support their role in reducing oxidative stress and improving behavioral outcomes [13, 22].

**Fig. 3:**
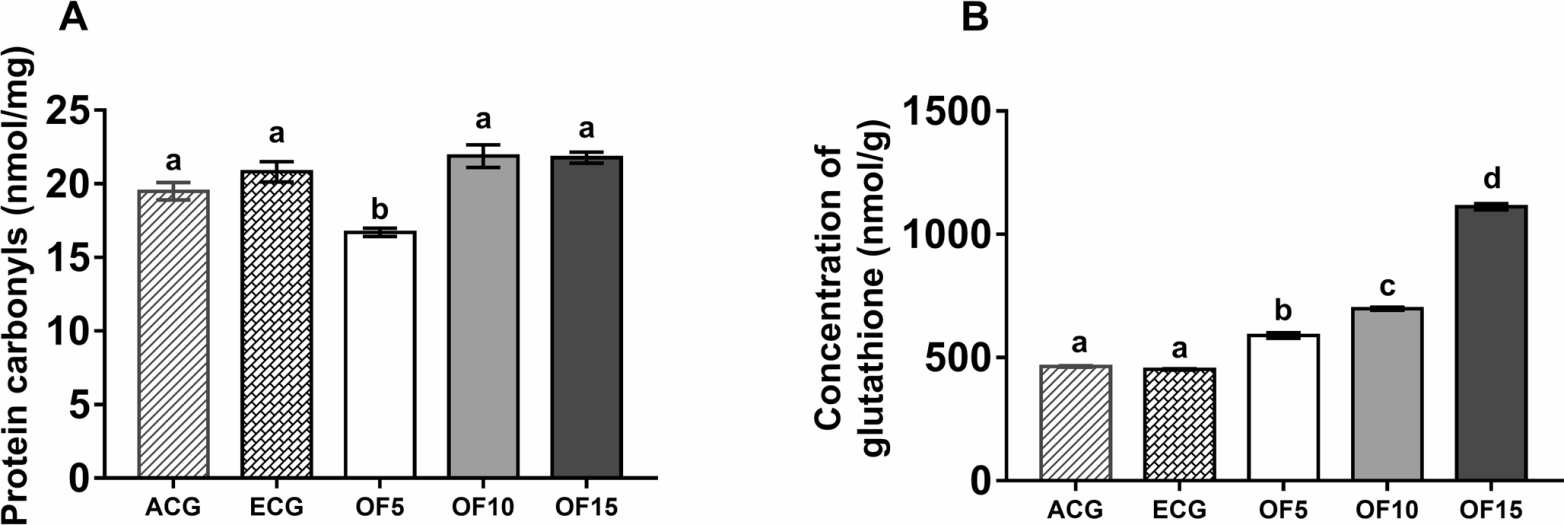
Concentration of brain glutathione and carbonyls in elderly rats treated with 5, 10 and 15% OFIF. Data are presented as mean ± standard deviation (One-way ANOVA, Holm-Sidak). Group sizes: ACG (Adult Control: animals fed a standard AIN-93 M diet *n* = 10), ECG (Elderly Control: animals fed a standard AIN-93 M diet *n* = 10), OF5 (animals fed a diet containing 5% cactus flour *n* = 10), OF10 (animals fed a diet containing 10% cactus flour *n* = 10), OF15 (animals fed a diet containing 15% cactus flour *n* = 10). Different letters between bars signify differences between groups (*p* < 0.05). (**A**) Protein carbonyls. (**B**) Concentration of glutathione

To assess oxidative status, we measured reduced glutathione (GSH) and protein carbonyls (PCO), a marker of protein oxidation [29]. PCO levels were significantly reduced in the OF5 group, while no differences were observed among control, OF10, and OF15 groups [F(4,25) = 17.45, *p* < 0.0001] (Fig. 3A). In contrast, GSH levels were higher in all treated groups compared to controls, with the highest levels in OF15 [F(4,25) = 1091, *p* < 0.0001] (Fig. 3B). Additionally, glutathione peroxidase (GPx) activity was increased in OFIF-treated groups.

Correlation analysis showed that PCO levels were positively associated with PWY-5884 (*r* = 0.94; *p* < 0.0001), while GSH levels correlated with *Prevotella* (*r* = 0.82), *Lachnospiraceae* (*r* = 0.92), and the RI index (*r* = 0.61) (*p* < 0.0001) Fig. 1 (available in the supplementary material). These findings suggest that OFIF enhances endogenous antioxidant defenses, improving the brain’s capacity to counteract oxidative stress.

Although OF10 and OF15 did not reduce PCO levels, the decrease observed in OF5 may indicate an early protective effect against protein oxidation. Overall, these results suggest that OFIF exerts dose-dependent and pathway-specific effects on oxidative stress rather than a strictly linear response in the aged brain [30].

## Fecal Microbiota

The fecal microbiota analysis, based on beta diversity, revealed significant differences among groups, with ACG showing the most distinct profile compared to the other treatments. Alpha diversity indices (Chao1 and Fisher) indicated increased richness in ACG and OF15 compared to ECG Fig. 2 (available in the supplementary material).

Beta diversity further confirmed that aging alters microbiota structure, characterized by reduced diversity and loss of beneficial taxa, potentially impairing gut-brain axis signaling [31]. In this context, OFIF appears as a promising strategy to restore microbial balance and support cognitive function.

Notably, OF15 supplementation partially restored microbial diversity, as shown by increased richness indices Fig. 3 (available in the supplementary material). *Opuntia ficus-indica* flour, rich in fermentable fibers such as pectin and mucilage, likely contributes to this effect by promoting SCFA production (acetate, propionate, and butyrate), which modulate microbial composition, strengthen intestinal barrier integrity, and regulate systemic and neuroinflammatory responses [13, 32]. These changes suggest a more functional and metabolically active intestinal environment, with implications for gut–brain axis communication.

**Fig. 4:**
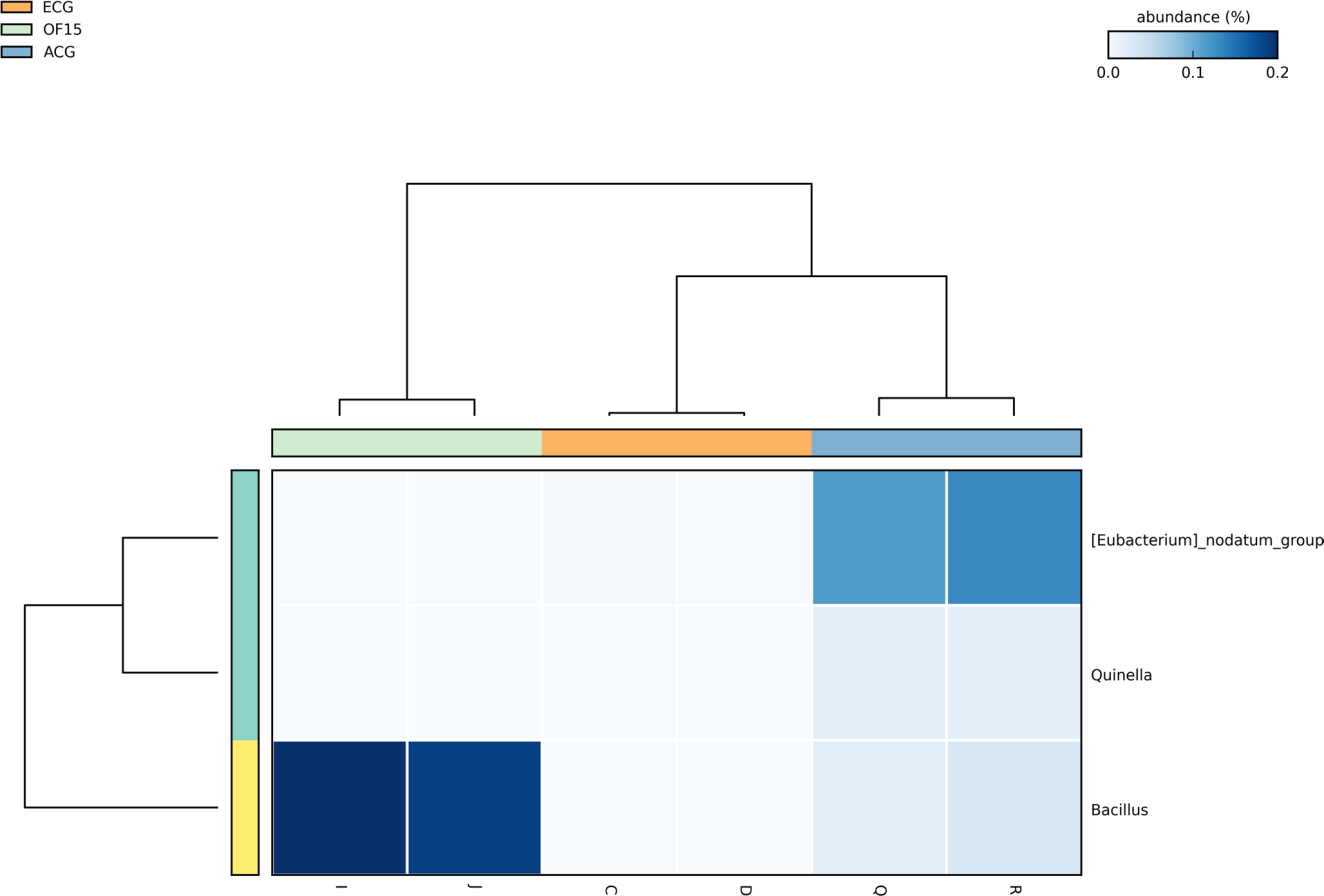
Differential abundance. The heatmap displays only the microbial genera that showed statistically significant differences in relative abundance among the three treatment groups

**Fig. 5:**
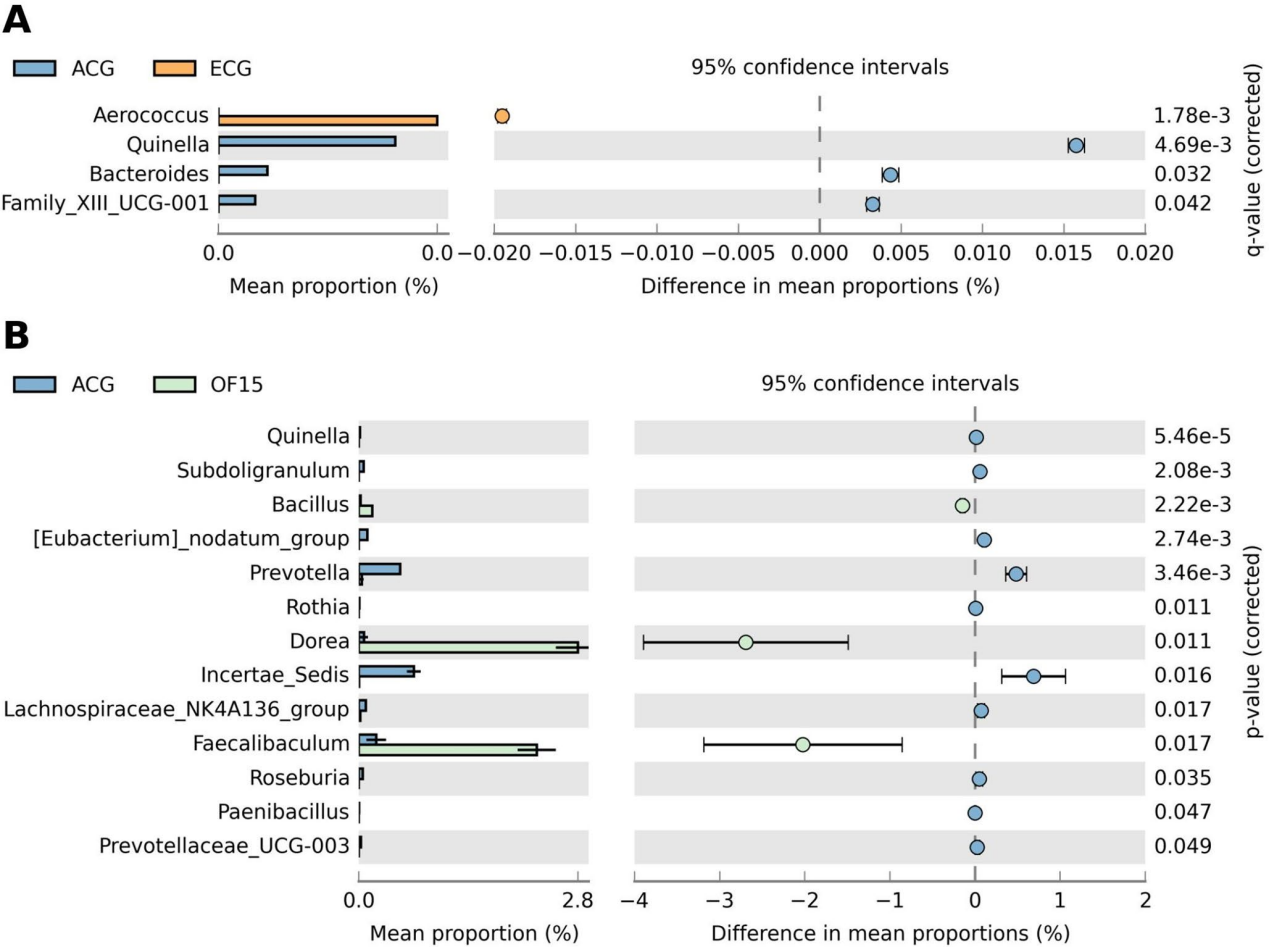
Differential abundance. (**A**) Comparison between the adult control group (ACG) and the elderly control group (ECG), highlighting genera with statistically significant differences in relative abundance. (**B**) Comparison between the adult control group (ACG) and the OF15-treated group, showing differentially abundant microbial genera between the two treatments. Only taxa with statistically significant differences are shown

Differential abundance analysis identified diet-dependent microbial shifts. Eubacterium nodatum was more abundant in ACG, while *Bacillus* was enriched in OF15 (Fig. 4). Comparisons also showed higher abundance of *Quinella*, *Bacteroides*, and *Family XII UCG-001* in ACG, and *Aerococcus* in ECG (Fig. 5A), indicating age-related variation. In contrast, OF15 showed enrichment of *Dorea*, *Bacillus*, and *Faecalibaculum* (Fig. 5B), along with *Quinella*, *Subdoligranulum*, *Prevotella*, *Rothia*, *Lachnospiraceae NK4A136*, *Roseburia*, *Paenibacillus*, and *Prevotellaceae UCG-003*, demonstrating strong microbiota modulation.

Taxonomically, OF15 enriched SCFA-producing such as *Faecalibaculum*, *Dorea*, *Prevotella*, and *Lachnospiraceae NK4A136*. These metabolites contribute to systemic and cerebral homeostasis by exerting anti-inflammatory effects, maintaining blood-brain barrier integrity, modulating neurotransmitters synthesis (e.g., Gamma-Aminobutyric Acid - GABA and serotonin), and promoting neurogenesis [33–35]. The increase in beneficial taxa and reduction of *Aerococcus* suggest a microbiota shift toward an anti-inflammatory and neuroprotective profile.

**Fig. 6:**
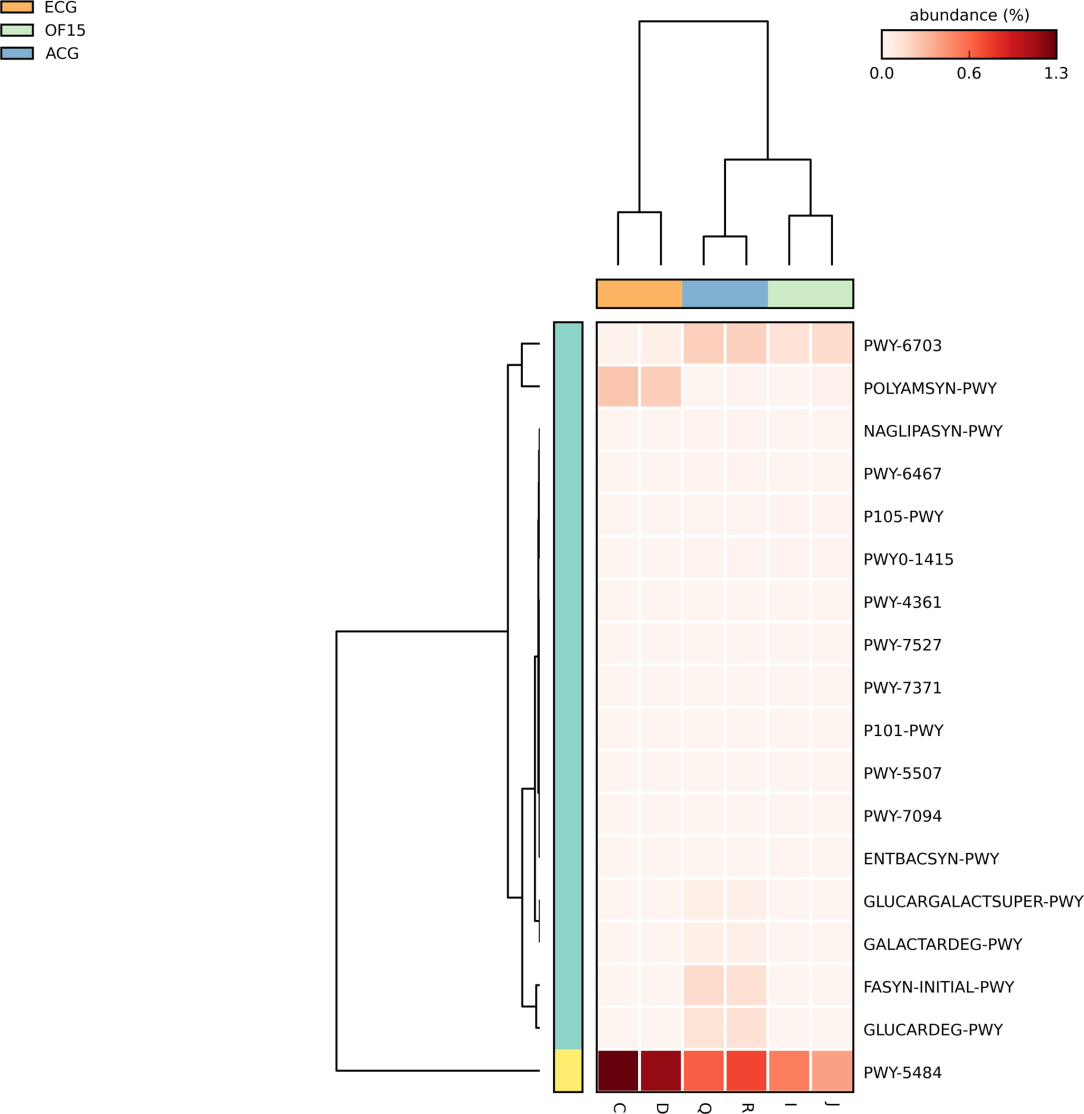
Functional prediction and representation of metabolic pathways with significantly different abundances among the three treatments, as identified by functional prediction analysis using PICRUS

In Fig. 6, metabolic pathway prediction showed reduced glycolysis (PWY-5484) in OF15, alongside increased activity in TCA cycle IV (P105-PWY), methionine salvage (PWY-7527), ribose degradation (PWY-4361), and enterobactin biosynthesis (ENTBACSYN-PWY) (Figs. 4, 5, 6, 7 and 8 are available in the supplementary material). These findings suggest a shift toward alternative energy utilization, including amino acid-derived carbon sources. Increased enterobactin biosynthesis may also influence host iron metabolism.

Cognitive improvements in OF15 animals, particularly in short- and long-term memory, were associated with a more balanced and metabolically active microbiota, reinforcing the role of the gut–brain axis in central nervous system modulation.

Despite these findings, limitations include the absence of central molecular markers (e.g., the brain-derived neurotrophic factor (BDNF), the cAMP response element-binding protein (CREB), and neurotransmitter), which restrict deeper mechanistic interpretation. Additionally, the specific bioactive compounds responsible for these effects remain unidentified. Future studies integrating phytochemical characterization, metabolomics and functional metagenomics are needed to clarify mechanisms and establish causal relationships between diet, microbiota, and neurobiological outcomes.

## Conclusions

This study provides novel evidence that dietary supplementation with *Opuntia ficus-indica* flour (OFIF), particularly in a 15% concentration, enhances cognitive performance and exerts neuroprotective effects in aged rats. These benefits were accompanied by improvements in brain lipid profiles, activation of endogenous antioxidant defenses, and partial restoration of both the composition and functional activity of the gut microbiota, suggesting a beneficial modulation of the gut–brain axis.

*Opuntia ficus-indica* flour thus exhibits promising nutraceutical potential and may be considered a viable dietary strategy to support cognitive health during aging. Nevertheless, translational studies in elderly human populations, along with appropriate extrapolation of the doses tested in animal models, are needed to confirm the safety and efficacy of this nutritional intervention.

## Supplementary Information

Below is the link to the electronic supplementary material.

Supplementary Material 1 (DOCX 2.57 MB)

Supplementary Material 2 (DOCX 449 KB)

Supplementary Material 3 (DOCX 2.00 MB)

## Data Availability

No datasets were generated or analysed during the current study.
