# Supplementary material for: Opuntia ficus-indica Flour Modulates Fecal Microbiota, Reduces Cerebral Oxidative Stress and Improves Cognitive Function in Elderly Rats
Source: Plant Foods Hum Nutr. 2026 May 7;81(2):51. doi: 10.1007/s11130-026-01510-3 (PMC13152895; doi:10.1007/s11130-026-01510-3)
Supplement: Supplementary file 1 — Supplementary Material 1 (DOCX 2.57 MB) [file 11130_2026_1510_MOESM1_ESM.docx]

**SUPPLEMENTARY MATERIAL**

***Opuntia ficus-indica* flour modulates fecal microbiota, reduces cerebral oxidative stress and improves cognitive function in elderly rats**

Renally de Lima Moura^1^*, Diego Elias Pereira^2,3,4^, Maria da Vitória Santos do Nascimento^5^, Larissa Maria Gomes Dutra^2,4^, Roberto Germano Costa^6^, Marcelo Sobral da Silva^7^, Josean Fechine Tavares^7^, Yuri Mangueira do Nascimento^7^, Vanessa Bordin Viera^2,3,4,8^, Juliano Carlo Rufino Freitas^9^, Wydemberg José de Araújo^10^, Fábio Anderson Pereira da Silva^1,11,12^, Valquiría Cardoso da Silva Ferreira^11,12^, Ariosvaldo Nunes de Medeiros^13^, Juliana Kessia Barbosa Soares^1,4^.

^1^ Food Science and Technology Program, Federal University of Paraíba, João Pessoa, PB, Brazil;

^2^ Laboratory of Experimental Nutrition, Department of Nutrition, Federal University of Campina Grande, Cuité, Brazil;

^3^ Center for Education and Health, Federal University of Campina Grande, Cuité, Brazil;

^4^ Post-Graduate Program in Natural Sciences and Biotechnology, Center for Education and Health, Federal University of Campina Grande, Cuité, Brazil;

^5^ Center for Medical Sciences, Graduate Program in Translational Health, Federal University of Pernambuco, Recife, PE, Brazil

^6^ Technologists Training Center - Campus IV, Department of Agriculture, Federal University of Paraíba, Brazil;

^7^ Post-Graduate Program in Bioactive Natural and Synthetic Products, Health Sciences Center, Federal University of Paraíba, João Pessoa, Brazil;

^8^ Laboratory for Synthesis and Analysis of Natural Antioxidants, Department of Nutrition, Federal University of Campina Grande, Cuité, CG, Brazil

^9^ Education and Health Center, Academic Unit of Biology and Chemistry, Federal University of Campina Grande, Cuité, CG, Brazil;

^10^ Federal Institute of Education, Science and Technology of Paraíba, Princesa Izabel, Brazil;

^11^ Chromatography and Spectrometry Laboratory, Department of Agroindustrial Management and Technology, Federal University of Paraíba, Bananeiras, Brazil;

^12^ Program in Agrifood Technology, Federal University of Paraíba, Bananeiras, Brazil;

^13^ Center for Agricultural Sciences - Campus III, Department of Animal Science, Federal University of Paraíba, Brazil;

*Corresponding author: E-mail: renally12moura@gmail.com - Phone: +55 83 99869-8024

**Plant Foods for Human Nutrition**

**1.Material and Methods**

**1.1 Collection of *Opuntia ficus-indica* Cladodes, Flour Processing, and Feed Preparation**

*Opuntia ficus-indica* cladodes were collected in March 2022 in the municipality of Bananeiras, Paraíba, Brazil (Latitude: 6° 45' 4'' S, Longitude: 35° 38' 0'' W). Young, healthy, and lesion-free cladodes were selected. The genetic material was registered in the Brazilian National System for the Management of Genetic Heritage and Associated Traditional Knowledge (SISGEN) under registration code A94A268. The other ingredients used for feed formulation were obtained from RHOSTER Indústria e Comércio LTDA and from local suppliers in the city of Cuité, Paraíba, Brazil.

Following collection, cladodes were manually, despined, sliced into 1 cm sections, and sanitized in a 200-ppm chlorine solution. They were then dried in a forced-air circulation oven (Biopar, model S480 CE, Porto Alegre – RS, Brazil) at 50 ± 1°C for 48 hours. The dried material was ground using a blender (Philco, model LQ PH900 PR, Brazil) and sieved through a 0.5 mm mesh to obtain a fine flour with uniform particle size. Subsequently, the feed was formulated based on the AIN-93M guidelines [1]. The composition of the experimental diets, containing 5%, 10%, and 15% *Opuntia ficus-indica* flour (OFIF), has been previously published in an earlier study conducted by our research group [2].

**1.2 Animals and experimental groups**

The study protocol was conducted in accordance with the ARRIVE Guidelines (Animal Research: Reporting of In Vivo Experiments) and was approved by the Ethics Committee on Animal Use (CEUA) of Unifacisa University Center, under protocol number 0.124.102.022. A total of 50 male Wistar rats were used, including ten adult rats aged 90–120 days (body weight: 250 ± 50 g) and 40 aged rats, 18 months old (body weight: 450 ± 50 g). The animals were randomly assigned to five groups (n = 10): Adult Control Group (ACG) and Elderly Control Group (ECG), which received the standard AIN-93M diet without OFIF ; and the experimental elderly groups OF5, OF10, and OF15, which received the AIN-93M diet containing 5%, 10%, and 15% *Opuntia ficus-indica* flour (OFIF), respectively. All animals were housed in collective polypropylene cages (three animals per cage; 34 cm wide, 49 cm long, and 16 cm high) under controlled environmental conditions (22 ± 1 °C, 65% relative humidity, and a 12-hour light/dark cycle starting at 6:00 a.m.). Feed and water were provided *ad libitum* throughout the four-week experimental period.

The formulation of the experimental diets was based on studies by Silva *et al.* [3], who administered the AIN-93M diet containing with 5%, 10%, and 15% wheat and oat bran to adult male Wistar rats, and Moura *et al.* [2], who used the same diet enriched with 5%, 10%, and 15% *Opuntia ficus-indica* flour in aged male rats of the same strain. The daily feed intake of 30 g was established according to [4] as described in the book Animal Nutrition: The Bases and Fundamentals of Animal Nutrition which recommends this amount as the standard average for laboratory rats. The duration of the treatment was determined based on the studies that investigated the antioxidant effects of purple sweet potato [5] and cactus flour consumption [2] in aged rats over a four-week period. The experimental design is illustrated in Figure 1.


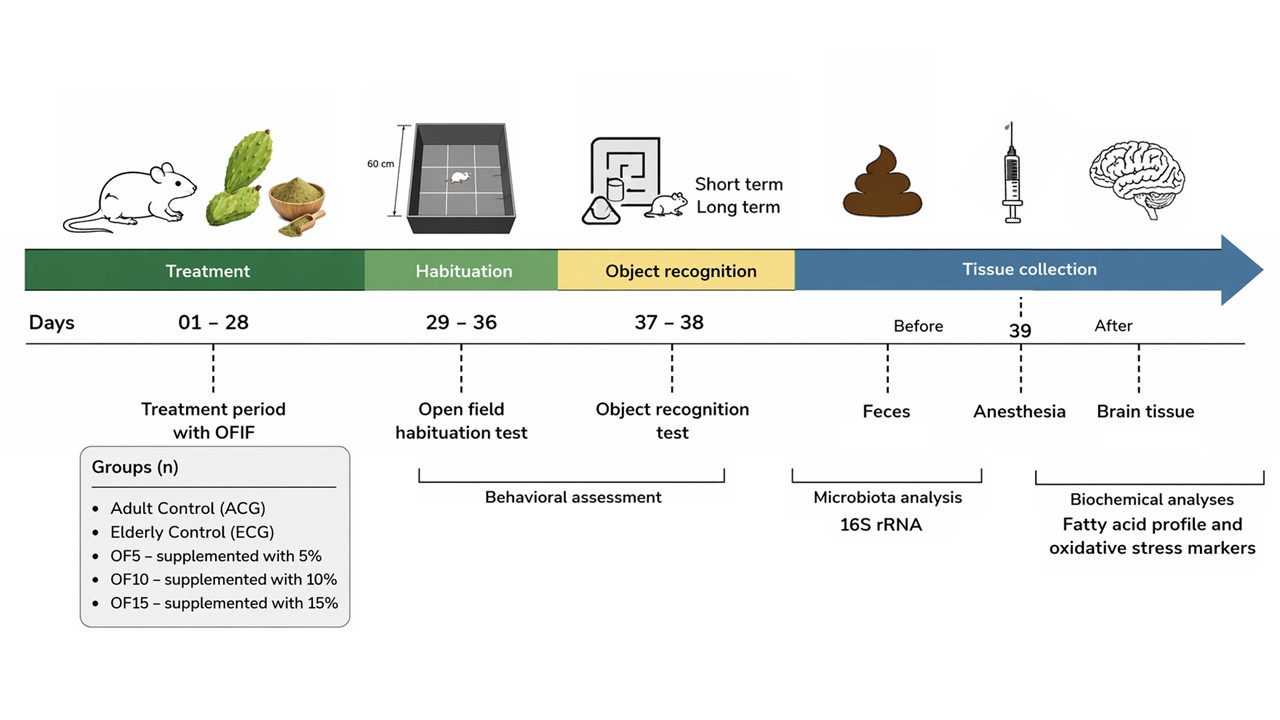


**Fig.1 Experimental Protocol.** Sequence of experimental days conducted with *Wistar* rats treated with a diet supplemented with OFIF. The groups included: Adult Control (ACG) and Elderly Control (ECG), which were fed the AIN-93M diet without OFIF, and the treated groups (OF5, OF10, OF15), which received diets supplemented with 5%, 10%, or 15% OFIF, respectively. The treatment was conducted from Day 1 to Day 28 (4 weeks). On Days 29 and 36, the open field habituation test was performed. On Days 37 and 38, the Object Recognition Test (ORT) was conducted to evaluate short and long-term memory. On Day 39, feces were collected exclusively from the ACG, ECG, and OF15 groups for microbiota analysis prior to anesthesia. After anesthesia, brain tissue was collected from all groups for evaluation of fatty acid profile and oxidative stress markers

**1.3 Behavioral test**

1.3.1 Assessment of non-associative learning

Adult and elderly animals were evaluated using the Open Field Habituation test and the Object Recognition Test (ORT). Each animal was exposed to the open field twice, in the first stage, the habituation test was performed and after 7 (seven) days, the same test was repeated in order to compare the locomotor activity of the animals for evaluation of non-associative learning [6]. The parameter analyzed through this test is the amount of explorative interactions taken by the animal to the field, considering the locomotion of the four legs toward the interior of each field. The test observation time was 10 min. The procedure was performed between 06:00 and 08:00 a.m., on each test day, and the sessions were filmed with a video camera. For each animal tested, the apparatus was cleaned with a 10 % alcohol solution before starting and after completion of the test.

1.3.2 Object recognition test (ORT)

To evaluate the short and long term memory, the Object Recognition Test (ORT) was used. The test was performed in the open field apparatus (60 × 60 × 60 cm), colored black, with six lines crossing forming 6–20 × 20 cm quadrants, uniformly lit, and with black color objects, with different shapes (rectangular or pyramid), and textures (smooth or rough) [7]. The test consisted of 4 (four) 10 min trials, taking place in 3 (three) steps: (1) Day 1–habituation for 10 min to minimize manipulation stress; (2) Day 2–performed 24 h after the habituation test, where each animal was placed in the open field containing two objects (FO1 and FO2) with identical textures (smooth), but with different forms (triangle and prismatic rectangle), located in two randomly chosen opposite corners. On the same day, yet 1 h later, the animal was placed in the open field again to explore two objects (FO1 in its original location, and a new object–NO1, identical to FO1 but with a different texture, and located in the place where FO2 had been placed during the habituation test; and (3) Day 3–was performed 24 h after the short duration test; each animal was placed in the open field to explore two objects (FO2 in its original place) and a new object (NO2) being identical to FO2 but with different texture. The parameters evaluated during the test sessions include the time (in seconds) spent exploring the new objects (TNO) and the time (in seconds) spent exploring the familiar objects (TFO). To assess the ability to recognize the objects, the following indices were calculated: recognition index (RI), calculated using the formula: RI = TNO / (TNO + TFO), which corresponds to the time spent exploring the new object divided by the total time spent exploring both objects; and the discrimination index (DI), calculated as the difference in exploration time between the new object and the familiar object divided by the total exploration time of both objects [DI = (TNO − TFO) / (TNO + TFO)] [8, 9, 10, 11]. For DI, the result could range from + 1 to − 1, with positive values indicating more time spent with the new object, and negative or near-zero values indicating low discrimination ability and, consequently, more time spent with the familiar object [12, 13, 14].

**1.4 Analysis of fecal microbiota using the 16S amplicon rRNA metagenome**

1.4.1 Sequencing of 16S rRNA gene

Individual stool samples from the animals were collected at adult (90 days of age) and elderly (547 days of age) groups and immediately stored at − 20 °C. Six samples from each group were used for DNA sequencing of the 16S rRNA gene. Genomic DNA was extracted using a commercial kit (PowerFecal Pro DNA, Qiagen, Hilden, Germany). DNA integrity was assessed by 1 % agarose gel electrophoresis and quantified by fluorometry (Qubit, ThermoFisher, Waltham, MA, USA). The V3-V4 regions of the microbial 16S rRNA gene were amplified by PCR (95 °C for 3 min, followed by 25 cycles at 95 °C for 30 s, 55 °C for 30 s, 72 °C for 30 s, and a final extension to 72 °C for 5 min) using primers 341F: 5′-TCG TCG GCA GCG TCA GAT GTG TAT AAG AGA CAG CCT ACG GGN GGC WGC AG-3′, and 785R: 5′-GTC TCG TGG GCT CGG AGA TGT GTA TAA GAG ACA GGA CTA CHV GGG TAT CTA ATC C-3′. The amplicon library was prepared using the Nextera XT Index Kit Set A (Illumina Inc., San Diego, CA, USA) and magnetic beads for cleaning and purification (Agencourt AMPureXP, Beckman Coulter, Indianapolis, USA). Paired-end sequencing was performed on the Illumina MiSeq platform using a 500 cycle V2 kit (2 × 250).

1.4.2 Bioinformatics analysis

The bioinformatics pipeline was used according to the recommendations of Liu et al.[15] and Liu et al. [16]. The raw demultiplexed Forward and Reverse sequences were processed in the QIIME 2 v.20.8 platform 30 [17]. Low quality reads were removed, with a Q > 20 minimum score for quality using the DADA2 program [18]. Chimeric and unsorted sequences were removed from the analysis as well as low quality bases above 245 bp. The generated Amplicon Sequence Variant (ASV) tables were normalized to 3150 ASVs per sample using DADA2. For phylogeny, the sequences were rarefied and aligned using the Silva 128 SEPP reference database available at https://docs.qiime2.org/2022.2/data-resources/. The alpha diversity analysis was evaluated using: Observed Features, Fisher, Simpson, and Shannon indices with QIIME 2 v.20.8 30 [17]. Taxonomic classifications were assigned by the Machine-learning method based on the Silva Databases with 99 % similarity for the V3-V4 regions.

1.4.3 Metabolic inference

Functional prediction of metagenomes was performed from 16S rRNA data using PICRUSt2 [19]. The abundance tables of ASVs generated through QIIME2 were used as input for PICRSt2 FullPipile through the q2-picrust2 plugin of QIIME2 available at https://library.qiime2.org/plugins/q2-picrust2/13/. Metabolic pathway and KO abundances were mapped using the MetaCyc database available at <https://metacyc.org/>.

**1.5** ​​**Brain Tissue Collection, Fatty Acid Profile Analysis, Protein Oxidation Product, and Glutathione**

On the day of euthanasia, following decapitation, the brains of animals from each group were removed, weighed using an ATX 224 analytical balance with internal calibration chamber (Shimadzu®), and stored at −20 °C for subsequent analysis of fatty acid ester composition (%) and protein oxidation.

1.5.1 Analysis of brain fatty acid profile

To determine the fatty acid profile of the brain the lipid extract of the products was first obtained using the method of Folch et al. [20]. From this extract, methyl esters were obtained by esterification following the methodology of Hartman and Lago [21]. The methyl esters were identified and quantified in a Ciola & Gregori Ltda gas chromatograph (model CG-Master), with a flame ionization detector. The chromatographic analysis used a polyethylene glycol column (Carbowax 20M), with fused silica, 30 m long, 0.53 mm in diameter, and 0.25 μm thick stationary phase film. The vaporizer and detector temperatures were 150 °C and 200 °C, respectively. The oven program was 80 °C for 30 min, with an increase of 10 °C /min up to 180 °C. The mobile phase was hydrogen, with a flow rate of 5 mL/min. A volume of 1 μL was injected, with a split ratio of 1:25. The characterization of fatty acids was carried out by comparing the mass spectrum obtained with standards also injected into GC–MS.

1.5.2 Analysis of Protein Oxidation in Brain Tissue

Protein oxidation was assessed by measuring the content of total carbonyl compounds, determined after sample derivatization with 2,4-dinitrophenylhydrazine (DNPH), according to the method proposed by Oliver et al. [22], with modifications. Approximately 1 g of brain tissue was homogenized in a 20 mM Na₃PO₄ buffer containing 0.6 M NaCl (1:10, pH 6.5) for 1 minute. A 150 μL aliquot was used for protein and carbonyl content determination. Proteins were precipitated with 1 mL of 10% trichloroacetic acid (TCA), followed by refrigerated centrifugation (4 °C) at 2400 g. For carbonyl compound determination, 1 mL of 0.2% DNPH in 2 M HCl was added to the sample. For the protein control, 1 mL of 2 M HCl was used instead. Samples were incubated in the dark for 1 hour. After incubation, proteins were precipitated again with 1 mL of 10% TCA and centrifuged at 9000 g for 10 minutes. The pellets were washed four times with 1 mL of ethanol/ethyl acetate solution (1:1, v/v), with centrifugation at 9000 g for 5 minutes between each wash. The final protein pellets were resuspended in 1.5 mL of 20 mM Na₃PO₄ buffer (pH 6.5) containing 6 M guanidine hydrochloride. Protein concentration was determined by measuring absorbance at 280 nm using a standard curve of bovine serum albumin (BSA). Carbonyl content was expressed as nmol of carbonyls per mg of protein, using a molar extinction coefficient of 21.0 nM⁻¹ cm⁻¹, with absorbance measured at 370 nm.

1.5.3 Determination of glutathione content of brain tissue

Total glutathione content was obtained by the method described by Anderson [23], and the results were expressed as nmol/g. Samples frozen with 1 mL of trichloroacetic acid solution were thawed and pricked with scissors for 15 s, then diluted to 1:20 (w/v) and homogenized for 2 min with an automatic homogenizer and centrifuged at 2000 G force at 4 °C for 5 min. The supernatants were then centrifuged at 9000 G force at 4 °C for 5 min. Supernatants were assayed for total glutathione.

**1.6 Statistical Analysis**

Results were expressed as mean ± SEM and analyzed by one-way ANOVA followed by Tukey’s post hoc test (p < 0.05). Prior to applying ANOVA, the Shapiro-Wilk test was used to assess data normality.

Associations between variables were assessed using a correlation matrix based on Spearman’s rank correlation coefficient. Correlations were considered relevant when the absolute correlation coefficient was ≥ 0.6 (|ρ| ≥ 0.6) and statistically significance (p ≤ 0.05), with 95% confidence intervals (95% CI). Statistical analyses were performed using GraphPad Prism® 8 (free version; GraphPad Software Inc., La Jolla, CA, USA).

For the analysis of fecal microbiota, raw reads in FASTQ format underwent a quality control process. Initially, during the demultiplexing and trimming steps, low-quality reads (PHRED score < Q30) were removed, as were the sequences with unsatisfactory lengths and chimeric reads, using UCHIME. The remaining high-quality reads were used in the definition of the Amplicon Sequence Variants (ASVs) using the DADA2 algorithm (version 1.16). Taxonomic classification was determined using the QIIME 2 platform (https://qiime2.org/), with reference to the SILVA 138 database (99% similarity) for the V3–V4 region. Functional metabolic prediction was carried out using PICRUSt2 (version 2.4.2) [24].

The diversity analysis, Alpha and Beta indices, and Core Microbiome were performed using MicrobiomeAnalyst 2.0 [25]. Taxonomic and functional analyses were achieved using STAMP statistical software version 2v 1.3 [26]. The Farthest Neighbor approach was applied for clustering analyses, while the ANOVA test was employed to compare multiple groups. Benjamini-Hochberg was employed as a multiple test correction, using p-value < 0.05 as a filter for statistical results. A two-sided t-test was used, and the Storey FDR correction method was applied for comparative analyses between two groups.

**References**

1. Reeves, P. G., Nielsen, F. H., & Fahey, G. C., Jr. (1993). AIN-93 purified diets for laboratory rodents: final report of the American Institute of Nutrition Ad Hoc writing committee on the reformulation of the AIN-76A rodent diet. J Nutr. 123:1939–1951.<https://doi.org/10.1093/jn/123.11.1939>
2. Moura, R. de L., Dutra, L. M. G., Nascimento, M. V. S. do, Oliveira, J. C. N. de, Viera, V. B., Dantas, B. S., Costa, R. G., Silva, M. S. da, Medeiros, A. N. de, Nascimento, Y. M. do, Tavares, J. F., & Soares, J. K. B. (2023). Cactus flour (Opuntia ficus-indica) reduces brain lipid peroxidation and anxious-like behavior in old Wistar rats. Physiol Behav. 272. [https://doi.org/10.1016/j.physbeh.2023.114360](https://doi.org/10.1016/j.physbeh.2023.114360?utm_source=chatgpt.com)
3. Silva, M. A. M. da, Barcelos, M. de F. P., Sousa, R. V. de, Lima, H. M., Falco, I. R., Lima, A. L. de, & Pereira, M. C. de A. (2003). Effect of wheat and oat bran fibers on the lipid profile of mouse (Rattus novergicus) Wistar blood samples. Ciênc. Agrotec. 27:6, 1321–1329
4. Andriguetto, J. M., Perly, L., Minardi, J., Souza, G. A., & Fialho, B. (1984). Nutrição animal: as bases e os fundamentos da nutrição animal: os alimentos. 2º ed. São Paulo: Nobel.
5. Shan, Q., Lu, J., Zheng, Y., Li, J., Zhou, Z., Hu, B., Zhang, Z., Fan, S., Mao, Z., Wang, Y.-J., & Ma, D. (2009). Purple sweet potato color ameliorates cognition deficits and attenuates oxidative damage and inflammation in aging mouse brain induced by D-Galactose. J Biomed Biotechnol. [https://doi.org/10.1155/2009/564737](https://doi.org/10.1155/2009/564737?utm_source=chatgpt.com)
6. Rachetti, A. L. F., Arida, R. M., Patti, C. L., Zanin, K. A., Fernandes-Santos, L., Frussa-Filho, R., Gomes da Silva, S., Scorza, F. A., & Cysneiros, R. M. (2013). Fish oil supplementation and physical exercise program: distinct effects on different memory tasks. Behav Brain Res. 237:283–289.<https://doi.org/10.1016/j.bbr.2012.09.048>
7. Nava-Mesa, M. O., Lamprea, M. R., & Múnera, A. (2013). Divergent short- and long-term effects of acute stress in object recognition memory are mediated by endogenous opioid system activation. Neurobiol Learn Mem. 106:185–192.<https://doi.org/10.1016/j.nlm.2013.09.002>
8. D’Avila, L. F., Dias, V. T., Vey, L. T., Milanesi, L. H., Roversi, K., Emanuelli, T., Bürger, M. E., Trevizol, F., & Maurer, H. L. (2017). Toxicological aspects of interesterified fat: Brain damages in rats. Toxicol Lett. 276:122–128. [https://doi.org/10.1016/j.toxlet.2017.05.020](https://doi.org/10.1016/j.toxlet.2017.05.020?utm_source=chatgpt.com)
9. Kim, W., Yim, H. S., Yoo, D. Y., Jung, H. Y., Kim, J. W., Choi, J. H., Yoon, Y. S., Kim, D. W., & Hwang, I. K. (2016). Dendropanax morbifera Léveille extract ameliorates cadmium-induced impairment in memory and hippocampal neurogenesis in rats. BMC Complement Med Ther. 16:452.<https://doi.org/10.1186/s12906-016-1435-z>
10. Lueptow, L. M. (2017). Novel object recognition test for the investigation of learning and memory in mice. J Vis Exp. 55718.<https://doi.org/10.3791/55718>
11. Nazir, N., Karim, N., Abdel-Halim, H., Khan, I., Wadood, S. F., & Nisar, M. (2018). Phytochemical analysis, molecular docking and antiamnesic effects of methanolic extract of Silybum marianum (L.) Gaertn seeds in scopolamine induced memory impairment in mice. J Ethnopharmacol. 210:198–208. [https://doi.org/10.1016/j.jep.2017.08.026](https://doi.org/10.1016/j.jep.2017.08.026?utm_source=chatgpt.com)
12. Lin, W.-S., Lo, J.-H., Yang, J.-H., Wang, H.-W., Fan, S.-Z., Yen, J.-H., & Wang, P.-Y. (2017). Ludwigia octovalvis extract improves glycemic control and memory performance in diabetic mice. J Ethnopharmacol. 207:211–219. [https://doi.org/10.1016/j.jep.2017.06.044](https://doi.org/10.1016/j.jep.2017.06.044?utm_source=chatgpt.com)
13. Nillert, N., Pannangrong, W., Umka Welbat, J., Chaijaroonkhanarak, W., Sripanidkulchai, K., & Sripanidkulchai, B. (2017). Neuroprotective effects of aged garlic extract on cognitive dysfunction and neuroinflammation induced by β-amyloid in rats. Nutrients. 9:1. [https://doi.org/10.3390/nu9010024](https://doi.org/10.3390/nu9010024?utm_source=chatgpt.com)
14. Paul, S., Modak, D., Dutta, S., Chaudhuri, T. K., & Bhattacharjee, S. (2019). Evaluation of the effectiveness of Acmella uliginosa (Sw.) Cass. flower methanolic extract in pain amelioration and memory impairment in the experimental rat models: Search for an alternative remedy over opioid painkillers. Pharmacogn Mag. 15(Suppl. 1), S335–S345.<https://doi.org/10.4103/pm.pm_71_19>
15. Liu, X., Mao, B., Gu, J., Wu, J., Cui, S., & Wang, G. (2021). Blautia—a new functional genus with potential probiotic properties? Gut Microbes. 12:1.<https://doi.org/10.1080/19490976.2021.1875796>
16. Liu, Y.-X., Qin, Y., Chen, T., Lu, M., Qian, X., Guo, X., & Bai, Y. (2021). A practical guide to amplicon and metagenomic analysis of microbiome data. Protein Cell. 12:315–330.<https://doi.org/10.1007/s13238-020-00724-8>
17. Bolyen, E., Rideout, J. R., Dillon, M. R., Bokulich, N. A., Abnet, C. C., Al-Ghalith, G. A., Alexander, H., Alm, E. J., Arumugam, M., Asnicar, F., Bai, Y., Bisanz, J. E., Bittinger, K., Brejnrod, A., Brislawn, C. J., Brown, C. T., Callahan, B. J., Caraballo-Rodríguez, A. M., Chase, J., Cope, E. K., … Caporaso, J. G. (2019). Reproducible, interactive, scalable and extensible microbiome data science using QIIME 2. Nat Biotechnol. 37:852–857.<https://doi.org/10.1038/s41587-019-0209-9>
18. Callahan, B. J., McMurdie, P. J., Rosen, M. J., Han, A. W., Johnson, A. J. A., & Holmes, S. P. (2016).DADA2: High-resolution sample inference from Illumina amplicon data. Nat Methods. 13:581–583.<https://doi.org/10.1038/nmeth.3869>
19. Langille, M. G. I., Zaneveld, J., Caporaso, J. G., McDonald, D., Knights, D., Reyes, J. A., Clemente, J. C., Burkepile, D. E., Vega Thurber, R. L., Knight, R., Beiko, R. G., & Huttenhower, C. (2013).Predictive functional profiling of microbial communities using 16S rRNA marker gene sequences. Nat Biotechnol. 31:814–821.<https://doi.org/10.1038/nbt.2676>
20. Folch, J., Lees, M., & Sloane Stanley, G. H. (1957). A simple method for the isolation and purification of total lipids. J Biol Chem. 226:497–509. [https://doi.org/10.1016/S0021-9258(18)64849-5](https://doi.org/10.1016/S0021-9258(18)64849-5?utm_source=chatgpt.com)
21. Hartman, L., & Lago, R. C. (1973). Rapid preparation of fatty acid methyl esters. Lab Pract. 22:475-6.
22. Oliver, C. N., Ahn, B., Moerman, E. J., Goldstein, S., & Stadtman, E. R. (1987). Age-related changes in oxidized proteins. J Biol Chem. 262:5488–5491. [https://doi.org/10.1016/S0021-9258(18)45598-6](https://doi.org/10.1016/S0021-9258(18)45598-6?utm_source=chatgpt.com)
23. Anderson, M. E. (1985). Determination of glutathione and glutathione disulfide in biological samples. Methods Enzymol. 113:548–555. [https://doi.org/10.1016/S0076-6879(85)13073-9](https://doi.org/10.1016/S0076-6879(85)13073-9?utm_source=chatgpt.com)
24. Douglas, G. M., Maffei, V. J., Zaneveld, J. R., Yurgel, S. N., Brown, J. R., Taylor, C. M., Huttenhower, C., & Langille, M. G. I. (2020).PICRUSt2 for prediction of metagenome functions. Nat Biotechnol. 38:685–688.<https://doi.org/10.1038/s41587-020-0548-6>
25. Lu, Y., Zhou, G., Ewald, J., Pang, Z., Shiri, T., & Xia, J. (2023). MicrobiomeAnalyst 2.0: comprehensive statistical, functional and integrative analysis of microbiome data. Nucleic Acids Res. 51(W1), W310–W318.<https://doi.org/10.1093/nar/gkad407>
26. Parks, D. H., Tyson, G. W., Hugenholtz, P., & Beiko, R. G. (2014). STAMP: statistical analysis of taxonomic and functional profiles. Bioinformatics. 30:3123–3124.<https://doi.org/10.1093/bioinformatics/btu494>
