# Supplementary material for: Opuntia ficus-indica Flour Modulates Fecal Microbiota, Reduces Cerebral Oxidative Stress and Improves Cognitive Function in Elderly Rats
Source: Plant Foods Hum Nutr. 2026 May 7;81(2):51. doi: 10.1007/s11130-026-01510-3 (PMC13152895; doi:10.1007/s11130-026-01510-3)

**SUPPLEMENTARY MATERIAL**

***Opuntia ficus-indica* flour modulates fecal microbiota, reduces cerebral oxidative stress and improves cognitive function in elderly rats**

Renally de Lima Moura^1^*, Diego Elias Pereira^2,3,4^, Maria da Vitória Santos do Nascimento^5^, Larissa Maria Gomes Dutra^2,4^, Roberto Germano Costa^6^, Marcelo Sobral da Silva^7^, Josean Fechine Tavares^7^, Yuri Mangueira do Nascimento^7^, Vanessa Bordin Viera^2,3,4,8^, Juliano Carlo Rufino Freitas^9^, Wydemberg José de Araújo^10^, Fábio Anderson Pereira da Silva^1,11,12^, Valquiría Cardoso da Silva Ferreira^11,12^, Ariosvaldo Nunes de Medeiros^13^, Juliana Kessia Barbosa Soares^1,4^.

^1^ Food Science and Technology Program, Federal University of Paraíba, João Pessoa, PB, Brazil;

^2^ Laboratory of Experimental Nutrition, Department of Nutrition, Federal University of Campina Grande, Cuité, Brazil;

^3^ Center for Education and Health, Federal University of Campina Grande, Cuité, Brazil;

^4^ Post-Graduate Program in Natural Sciences and Biotechnology, Center for Education and Health, Federal University of Campina Grande, Cuité, Brazil;

^5^ Center for Medical Sciences, Graduate Program in Translational Health, Federal University of Pernambuco, Recife, PE, Brazil

^6^ Technologists Training Center - Campus IV, Department of Agriculture, Federal University of Paraíba, Brazil;

^7^ Post-Graduate Program in Bioactive Natural and Synthetic Products, Health Sciences Center, Federal University of Paraíba, João Pessoa, Brazil;

^8^ Laboratory for Synthesis and Analysis of Natural Antioxidants, Department of Nutrition, Federal University of Campina Grande, Cuité, CG, Brazil

^9^ Education and Health Center, Academic Unit of Biology and Chemistry, Federal University of Campina Grande, Cuité, CG, Brazil;

^10^ Federal Institute of Education, Science and Technology of Paraíba, Princesa Izabel, Brazil;

^11^ Chromatography and Spectrometry Laboratory, Department of Agroindustrial Management and Technology, Federal University of Paraíba, Bananeiras, Brazil;

^12^ Program in Agrifood Technology, Federal University of Paraíba, Bananeiras, Brazil;

^13^ Center for Agricultural Sciences - Campus III, Department of Animal Science, Federal University of Paraíba, Brazil;

*Corresponding author: E-mail: renally12moura@gmail.com - Phone: +55 83 99869-8024

**Plant Foods for Human Nutrition**

**Multiple Group Analysis**

**Fig. 1 (SM):** Multiple Group Analysis. Hierarchical grouping based on Pearson's correlation matrix between behavioral and biological parameters in aged rats treated with cactus flour (*Opuntia ficus-indica*). Significant correlations were determined based on an r > 0.6 and p < 0.05. Positive and negative correlations are shown as red and blue, respectively.


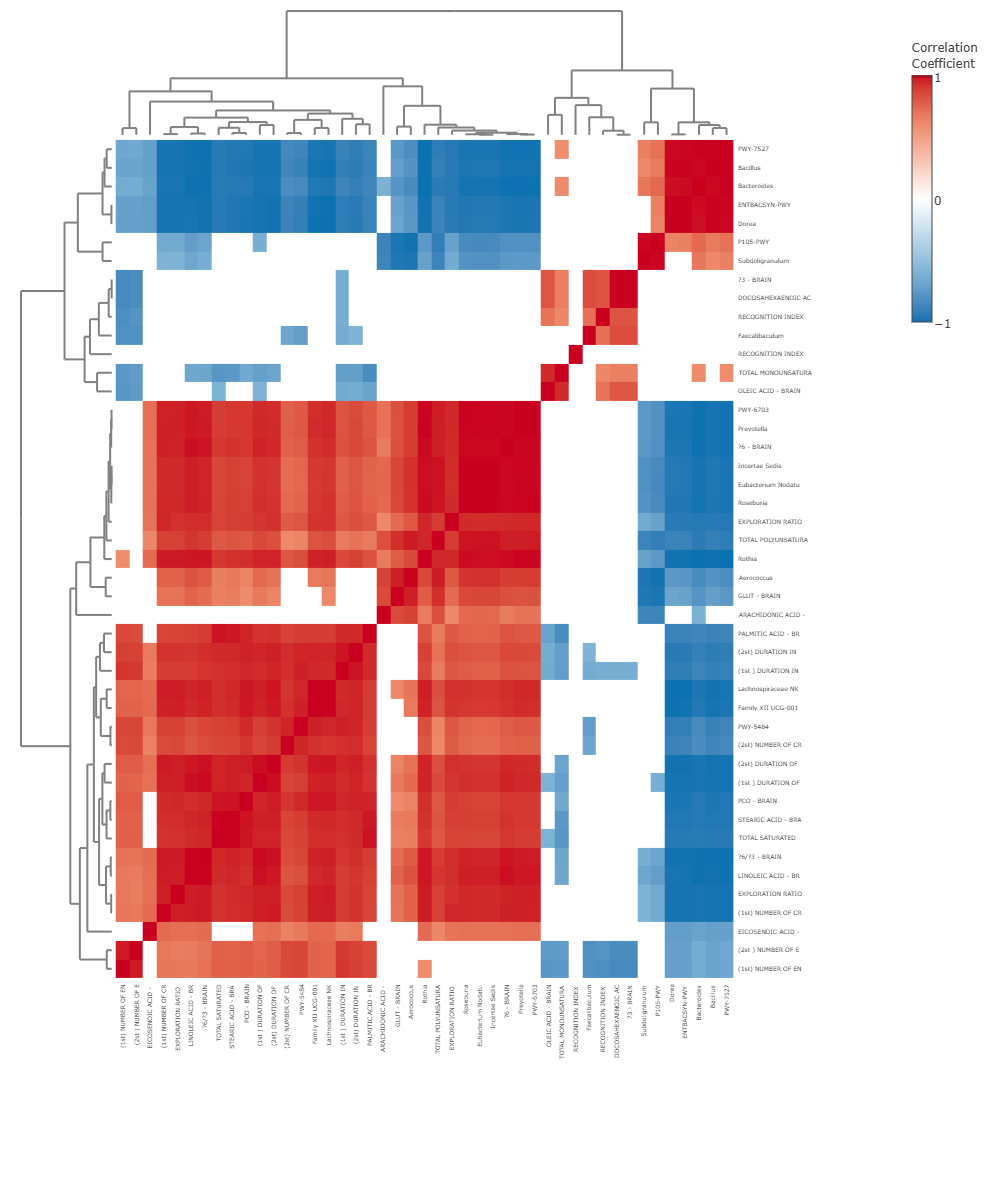


**Fig. 2 (SM) -** Beta diversity analysis. Microbial composition analysis was carried out using Principal Coordinates Analysis (PCoA) based on Bray–Curtis dissimilarity index. Significant differences were detected between treatment groups [PERMDISP] (F-value: 1.5053e+29; p-value: 3.1455e-44). Blue = ACG (Adult Control: animals fed a standard AIN-93M diet n = 10); Green = OF15 (animals fed a diet containing 15% cactus flour n = 10); Orange = ECG (Elderly Control: animals fed a standard AIN-93M diet n = 10).


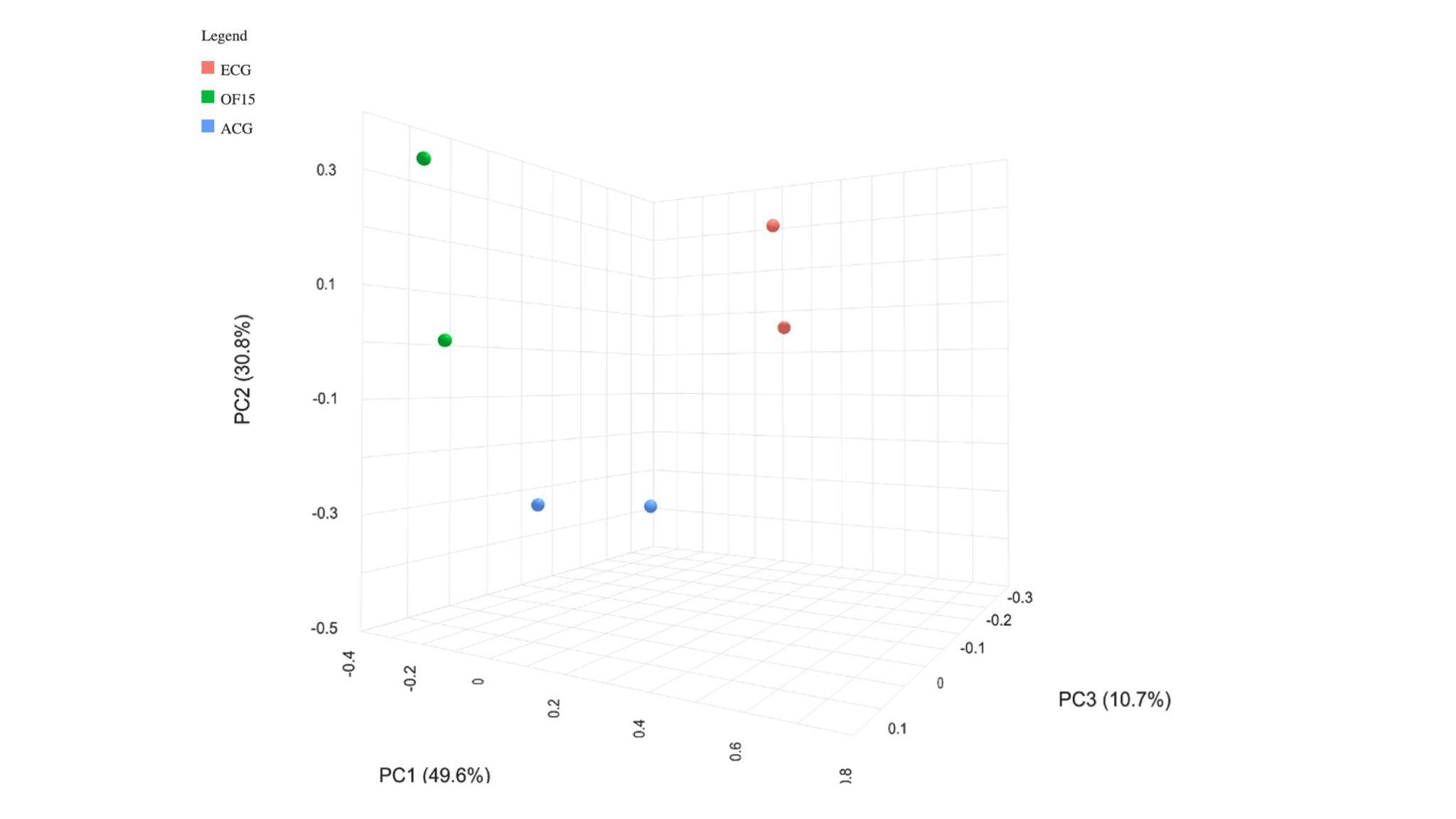


**Fig. 3 (SM) -** Alpha diversity. Alpha diversity values calculated using different indices are presented for the three treatment groups.


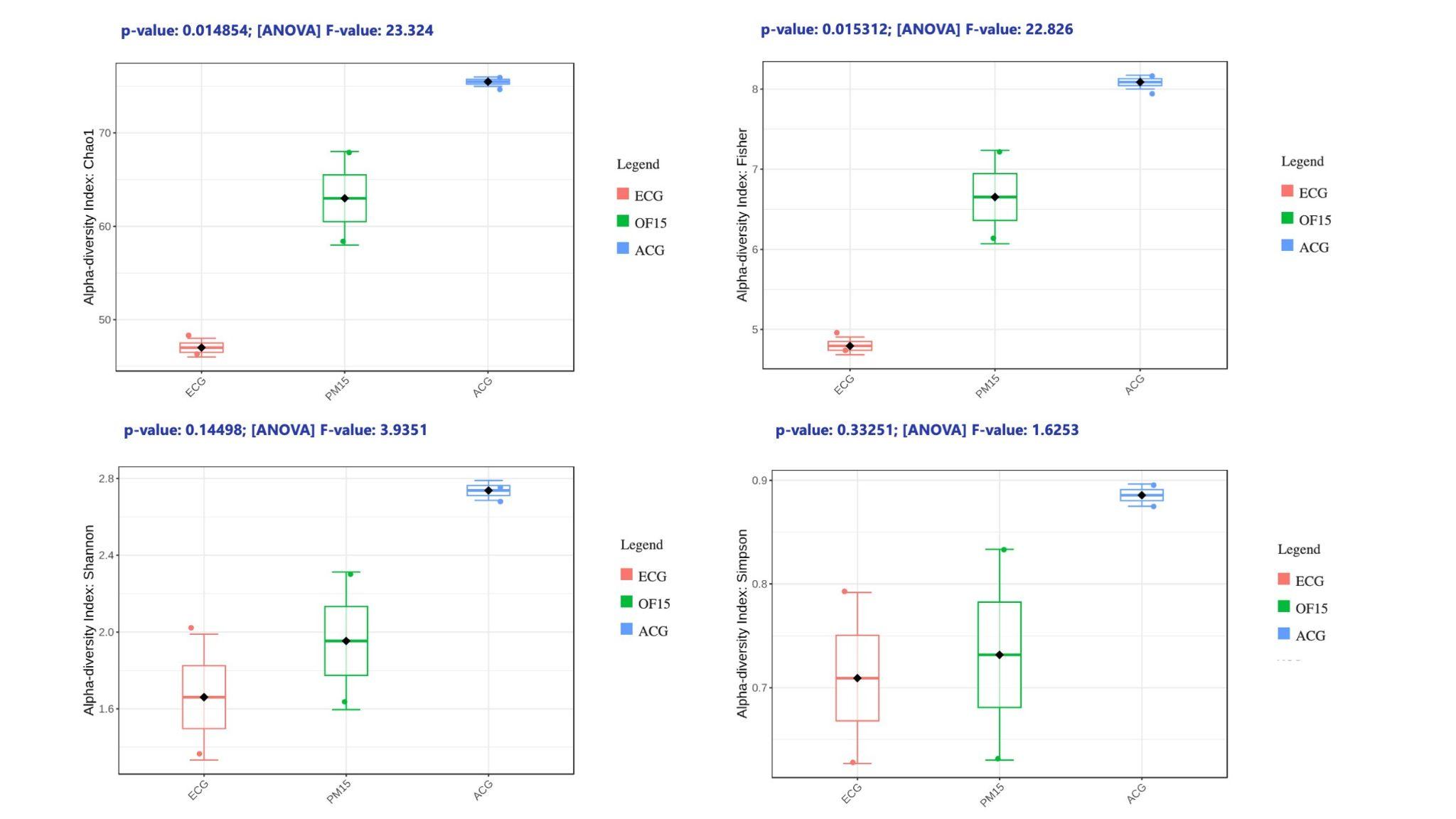


**Fig. 4 (SM) -** Differential abundance of the enterobactin biosynthesis pathway (ENTBACSYN-PWY) between groups.


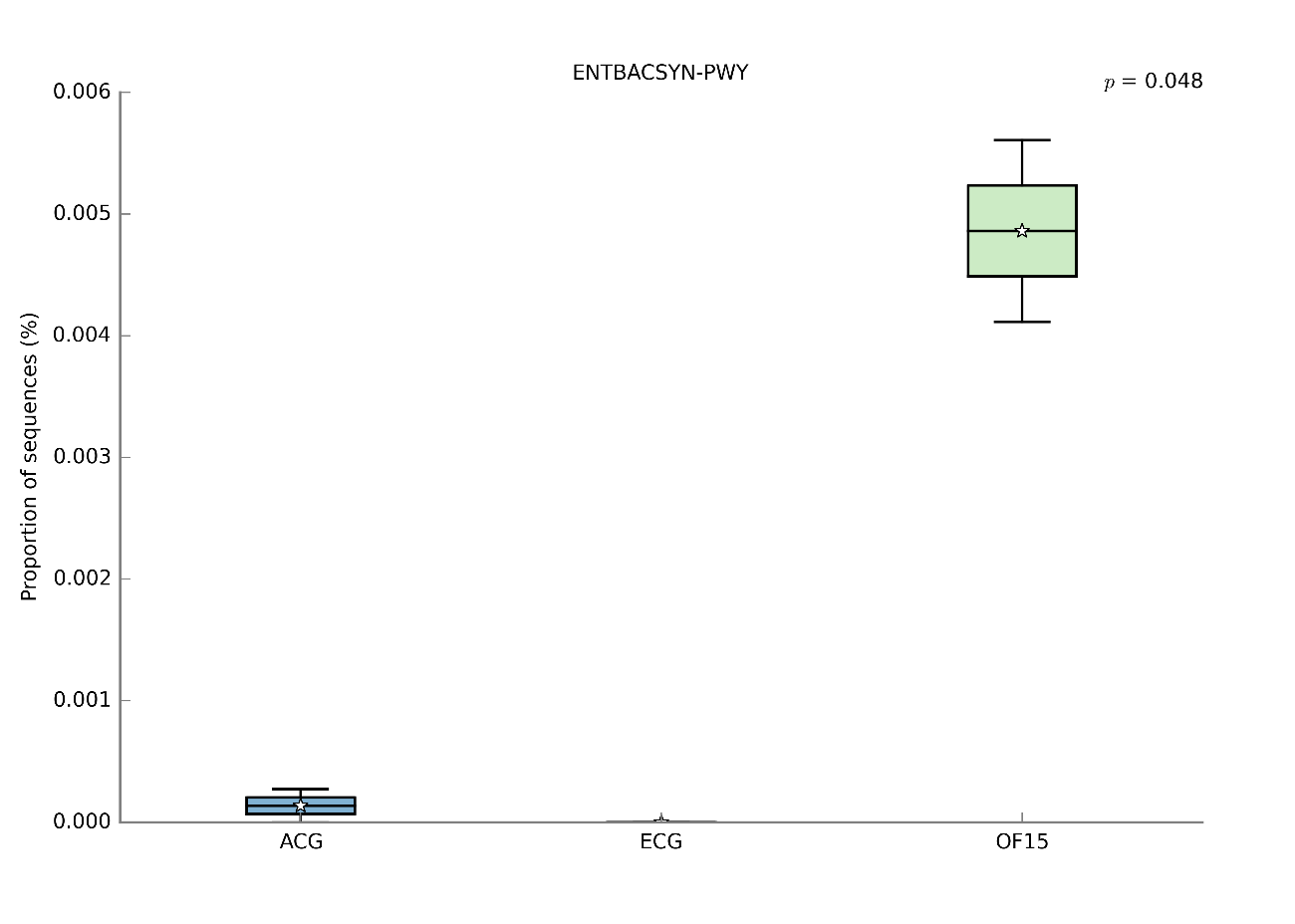


**Fig. 5 (SM) -** Differential abundance of the tricarboxylic acid (TCA) cycle IV – 2-oxoglutarate decarboxylase pathway (P105-PWY) between groups.


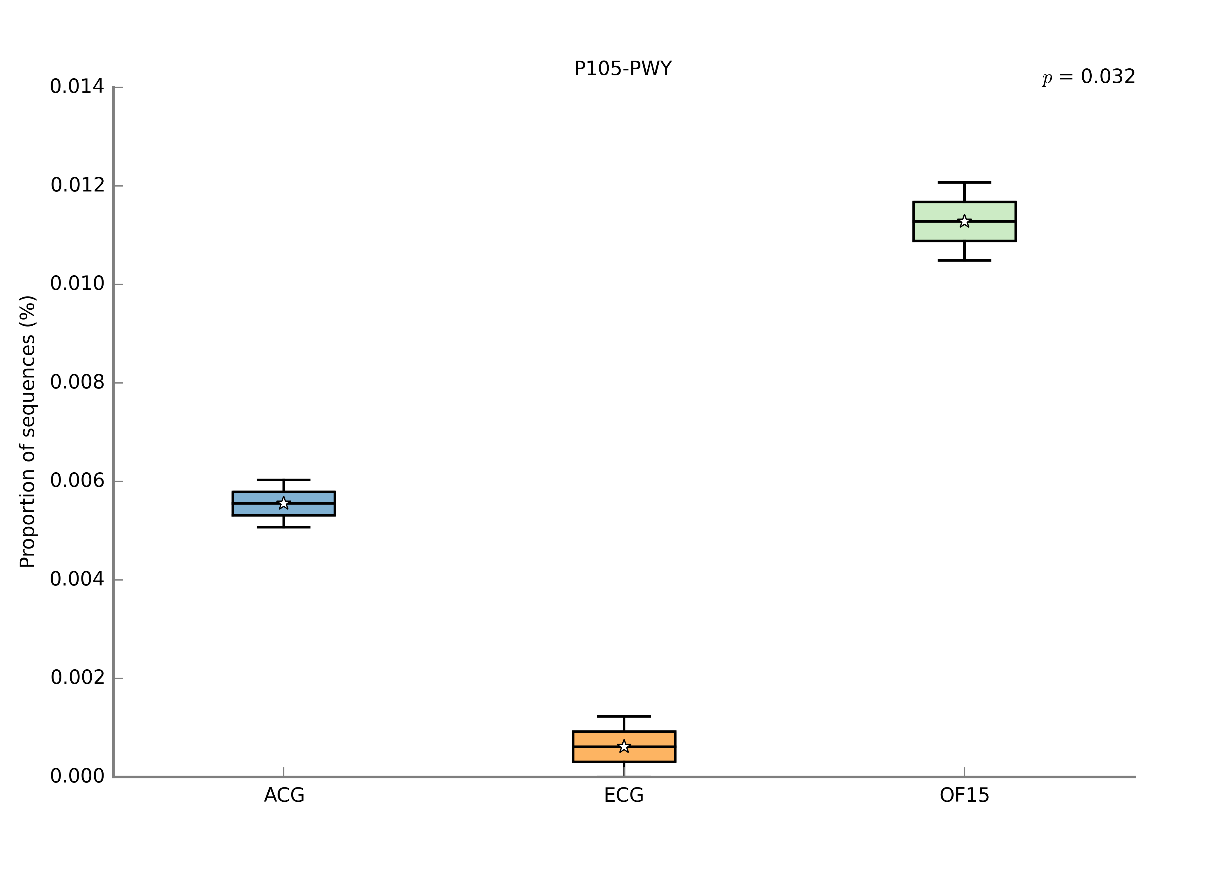


**Fig. 6 (SM) -** Differential abundance of the glycolytic pathway (PWY-5484) between groups.


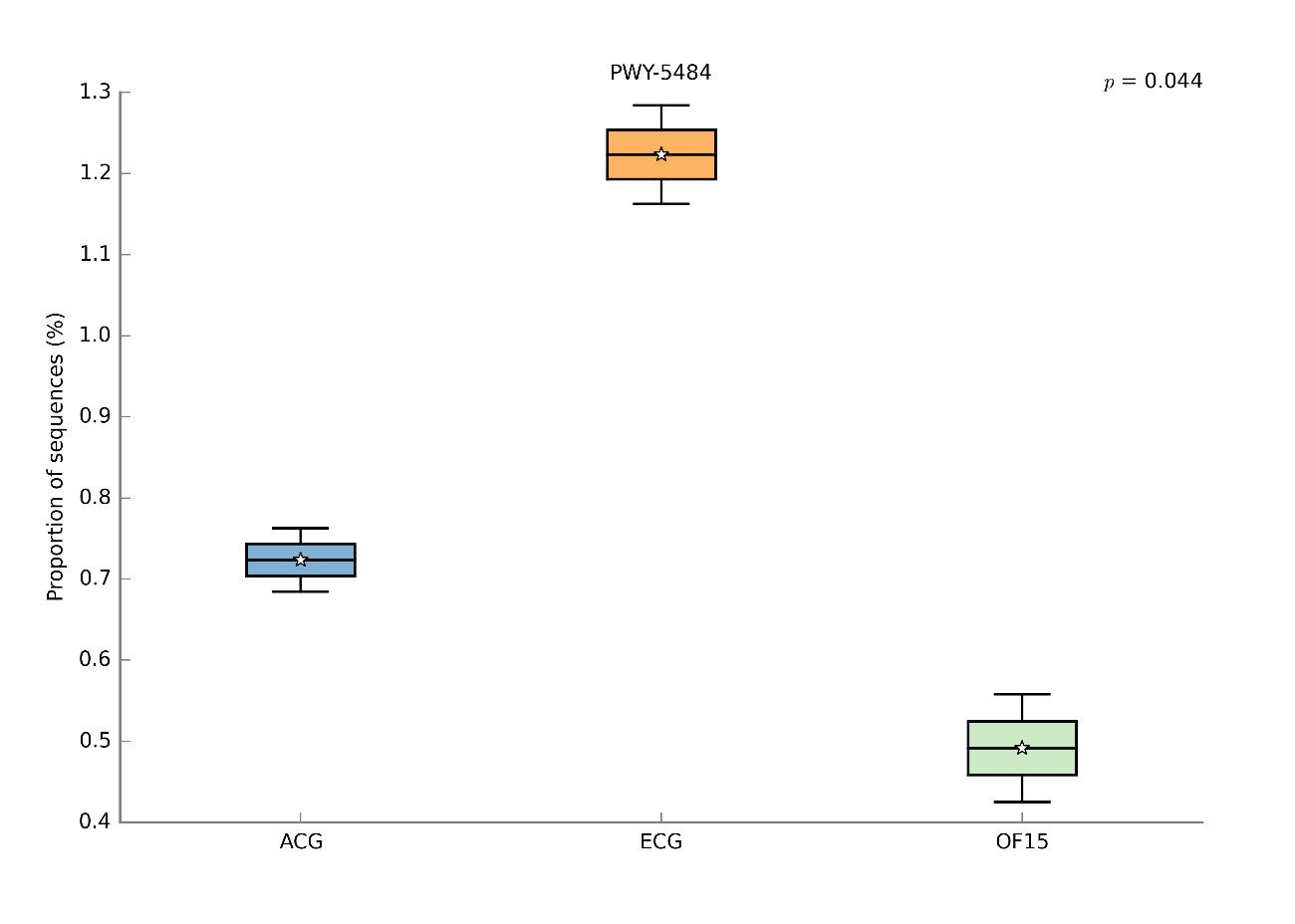


**Fig. 7 (SM) -** Differential abundance of the degradation of S-methyl-5-thio-α-D-ribose 1-phosphate pathway (PWY-4361) between groups.


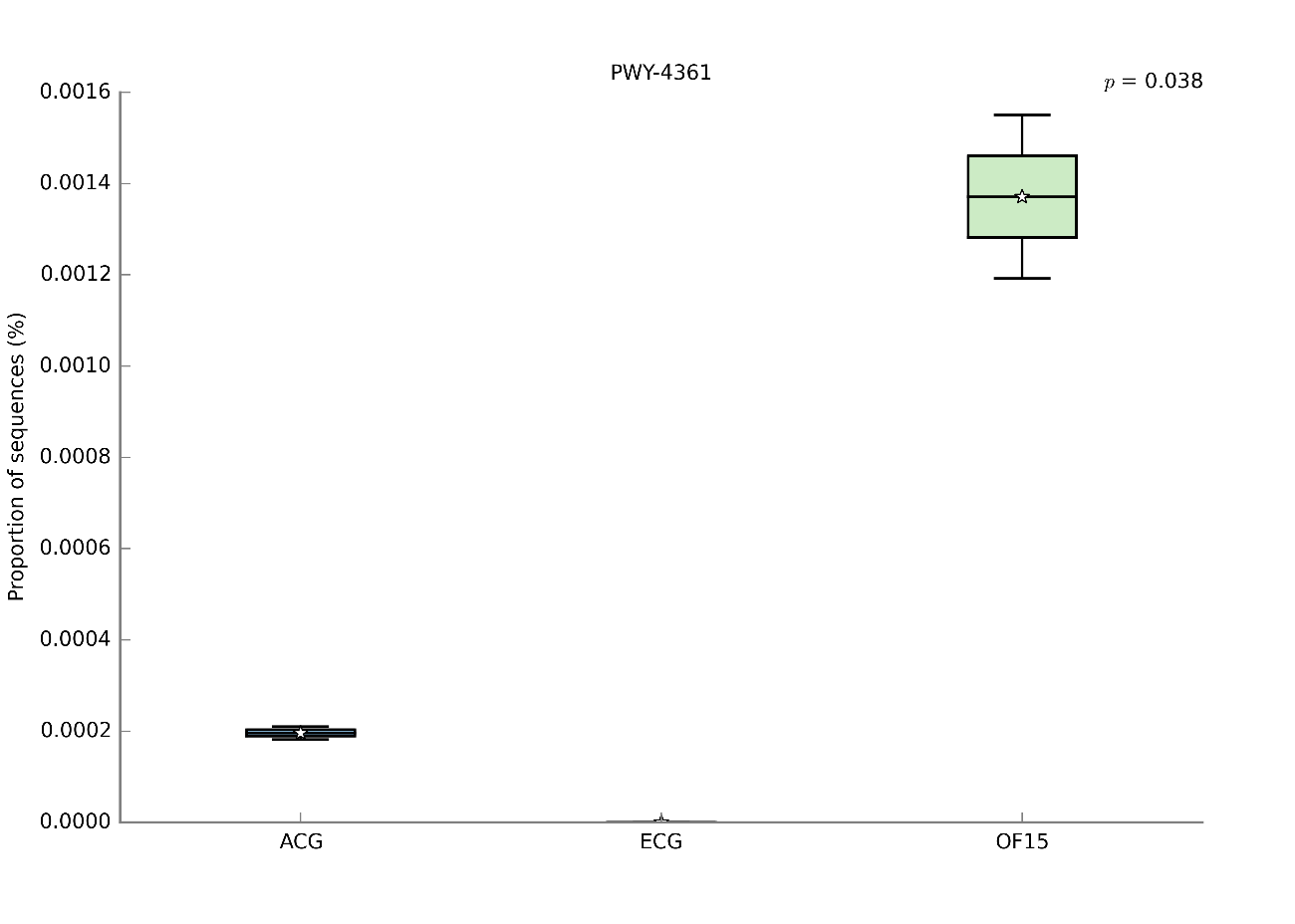


**Fig. 8 (SM) -** Differential abundance of the degradation of cyclic L-methionine salvage pathway III pathway (PWY-7527) between groups.


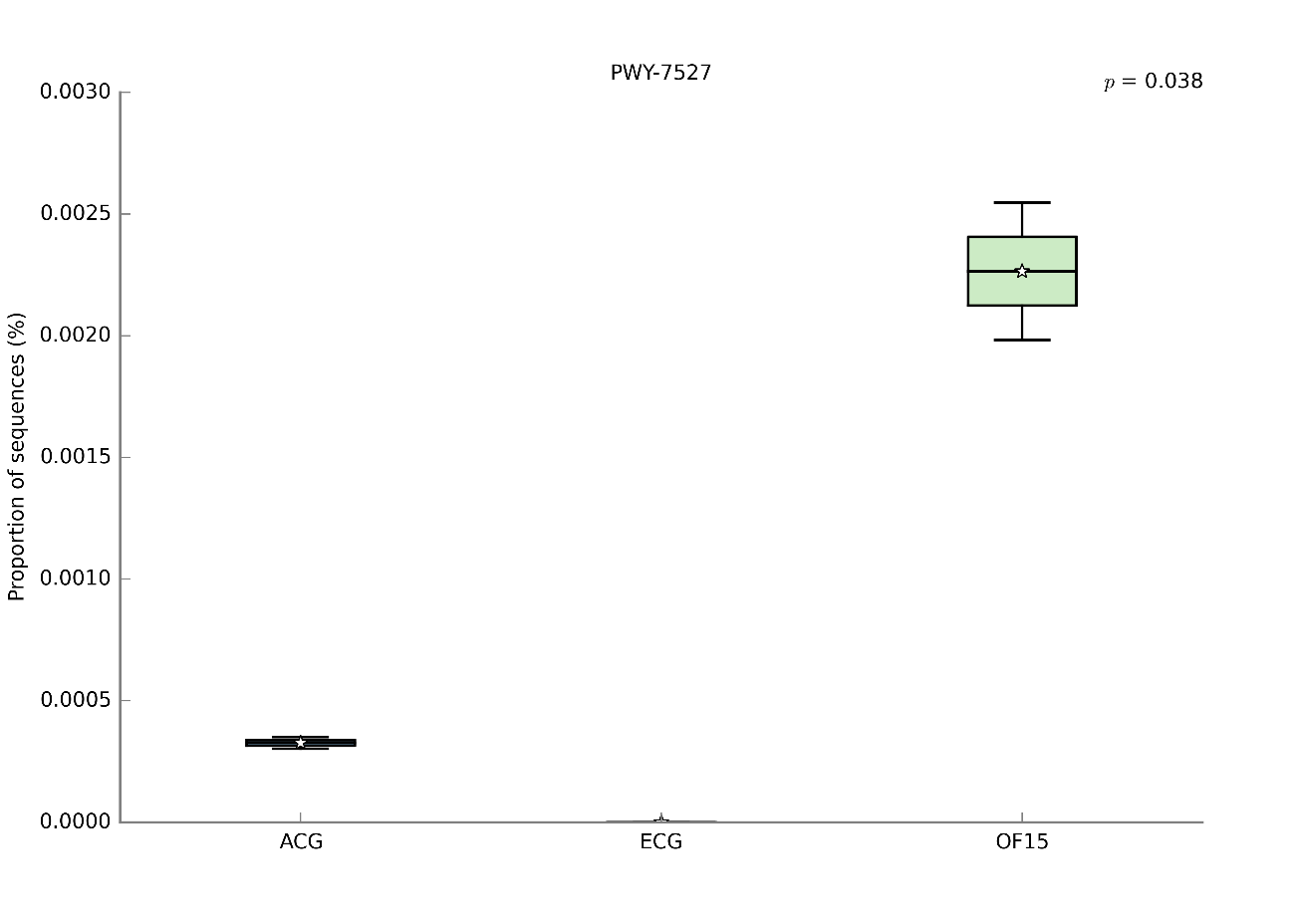

Supplement: Supplementary file 2 — Supplementary Material 2 (DOCX 449 KB) [file 11130_2026_1510_MOESM2_ESM.docx]
