# Supplementary material for: Opuntia ficus-indica Flour Modulates Fecal Microbiota, Reduces Cerebral Oxidative Stress and Improves Cognitive Function in Elderly Rats
Source: Plant Foods Hum Nutr. 2026 May 7;81(2):51. doi: 10.1007/s11130-026-01510-3 (PMC13152895; doi:10.1007/s11130-026-01510-3)
Supplement: Supplementary file 3 — Supplementary Material 3 (DOCX 2.00 MB) [file 11130_2026_1510_MOESM3_ESM.docx]

**SUPPLEMENTARY MATERIAL**

***Opuntia ficus-indica* flour modulates fecal microbiota, reduces cerebral oxidative stress and improves cognitive function in elderly rats**

Renally de Lima Moura^1^*, Diego Elias Pereira^2,3,4^, Maria da Vitória Santos do Nascimento^5^, Larissa Maria Gomes Dutra^2,4^, Roberto Germano Costa^6^, Marcelo Sobral da Silva^7^, Josean Fechine Tavares^7^, Yuri Mangueira do Nascimento^7^, Vanessa Bordin Viera^2,3,4,8^, Juliano Carlo Rufino Freitas^9^, Wydemberg José de Araújo^10^, Fábio Anderson Pereira da Silva^1,11,12^, Valquiría Cardoso da Silva Ferreira^11,12^, Ariosvaldo Nunes de Medeiros^13^, Juliana Kessia Barbosa Soares^1,4^.

^1^ Food Science and Technology Program, Federal University of Paraíba, João Pessoa, PB, Brazil;

^2^ Laboratory of Experimental Nutrition, Department of Nutrition, Federal University of Campina Grande, Cuité, Brazil;

^3^ Center for Education and Health, Federal University of Campina Grande, Cuité, Brazil;

^4^ Post-Graduate Program in Natural Sciences and Biotechnology, Center for Education and Health, Federal University of Campina Grande, Cuité, Brazil;

^5^ Center for Medical Sciences, Graduate Program in Translational Health, Federal University of Pernambuco, Recife, PE, Brazil

^6^ Technologists Training Center - Campus IV, Department of Agriculture, Federal University of Paraíba, Brazil;

^7^ Post-Graduate Program in Bioactive Natural and Synthetic Products, Health Sciences Center, Federal University of Paraíba, João Pessoa, Brazil;

^8^ Laboratory for Synthesis and Analysis of Natural Antioxidants, Department of Nutrition, Federal University of Campina Grande, Cuité, CG, Brazil

^9^ Education and Health Center, Academic Unit of Biology and Chemistry, Federal University of Campina Grande, Cuité, CG, Brazil;

^10^ Federal Institute of Education, Science and Technology of Paraíba, Princesa Izabel, Brazil;

^11^ Chromatography and Spectrometry Laboratory, Department of Agroindustrial Management and Technology, Federal University of Paraíba, Bananeiras, Brazil;

^12^ Program in Agrifood Technology, Federal University of Paraíba, Bananeiras, Brazil;

^13^ Center for Agricultural Sciences - Campus III, Department of Animal Science, Federal University of Paraíba, Brazil;

*Corresponding author: E-mail: renally12moura@gmail.com - Phone: +55 83 99869-8024

**Plant Foods for Human Nutrition**

**Table 1.** Fatty acid composition in the brain tissue of elderly Wistar rats treated with 5%, 10%, and 15% cactus flour (*Opuntia ficus-indica*).

| Fatty acids | Groups | | | |  |
| --- | --- | --- | --- | --- | --- |
|  |  | | | |  |
|  | **ACG** | **ECG** | **OF5** | **OF10** | **OF15** |
| SATURATED |  | |  | |  |
| Palmitic acid C16:0 | 15.4 ± 0.01^a^ | 13.6 ± 0.02^b^ | 6.23 ± 0.01^c^ | 13.92± 0.01^b^ | 14.21± 0.02^b^ |
| Stearic acid C18:0 | 15.25 ± 0.01^a^ | 13.52± 0.03^b^ | 8.23± 0.02^c^ | 14.10± 0.01^b^ | 15.12± 0.01^a^ |
| Total | **30.65** | **27.15** | **14.46** | **28.02** | **29.33** |
| MONOUNSATURATED |  |  |  |  |  |
| Oleic acid C18:1ω9 | 3.07± 0.01^a^ | 6.17± 0.02^b^ | 12.67± 0.01^c^ | 13.10 ± 0.01^c^ | 13.54± 0.02^c^ |
| Eicosenoic acid C20:1ω9 | 1.45± 0.01^a^ | 2.48 ± 0.02^b^ | 4.52 ± 0.01^c^ | 1.18 ± 0.02^a^ | 3.12 ± 0.03^b^ |
| Total | **4.52** | **8.65** | **17.19** | **14.28** | **16.66** |
| POLYUNSATURATED |  |  |  |  |  |
| Linoleic acid C18:2ω6c | 14.3 ± 0.02^a^ | 0.60± 0.03^b^ | 0.72± 0.02^c^ | 0.56± 0.01^b^ | 0.54± 0.01^b^ |
| Arachidonic acid C20:4ω6 | 6.31± 0.01^a^ | 5.53± 0.02^a^ | 10.40± 0.02^b^ | 10.92± 0.01^b^ | 14.48± 0.02^c^ |
| Docosahexaenoic acid C22:6ω3 | 4.74± 0.02^a^ | 5.87± 0.01^a^ | 12.50± 0.02^b^ | 14.63± 0.01^b^ | 20.87± 0.01^c^ |
| Total | **25.35** | **12.00** | **23.62** | **26.11** | **35,89** |
| SUMS |  |  |  |  |  |
| ω3 | 4.74 | 5.87 | 12.50 | 14.63 | 20.87 |
| ω6 | 20.61 | 6.13 | 11.12 | 11.48 | 15.02 |
| ω6/ω3 | 4.35 | 1.04 | 0.89 | 0.78 | 0.72 |

ACG = Adult Control: animals fed a standard AIN-93M diet; ECG = Elderly Control: animals fed a standard AIN-93M diet; OF5 = animals fed a diet containing 5% cactus flour; OF10 = animals fed a diet containing 10% cactus flour; OF15 = animals fed a diet containing 15% cactus flour. Values are expressed as mean ± standard deviation. Different superscript letters indicate statistically significant differences between groups (P < 0.05)
